# A Bioinformatics Guide to Plant Microbiome Analysis

**DOI:** 10.3389/fpls.2019.01313

**Published:** 2019-10-23

**Authors:** Rares Lucaciu, Claus Pelikan, Samuel M. Gerner, Christos Zioutis, Stephan Köstlbacher, Harald Marx, Craig W. Herbold, Hannes Schmidt, Thomas Rattei

**Affiliations:** Department of Microbiology and Ecosystem Science, University of Vienna, Vienna, Austria

**Keywords:** plant, microbiome, holobiont, omics, computational, experimental

## Abstract

Recent evidence for intimate relationship of plants with their microbiota shows that plants host individual and diverse microbial communities that are essential for their survival. Understanding their relatedness using genome-based and high-throughput techniques remains a hot topic in microbiome research. Molecular analysis of the plant holobiont necessitates the application of specific sampling and preparatory steps that also consider sources of unwanted information, such as soil, co-amplified plant organelles, human DNA, and other contaminations. Here, we review state-of-the-art and present practical guidelines regarding experimental and computational aspects to be considered in molecular plant–microbiome studies. We discuss sequencing and “omics” techniques with a focus on the requirements needed to adapt these methods to individual research approaches. The choice of primers and sequence databases is of utmost importance for amplicon sequencing, while the assembly and binning of shotgun metagenomic sequences is crucial to obtain quality data. We discuss specific bioinformatic workflows to overcome the limitation of genome database resources and for covering large eukaryotic genomes such as fungi. In transcriptomics, it is necessary to account for the separation of host mRNA or dual-RNAseq data. Metaproteomics approaches provide a snapshot of the protein abundances within a plant tissue which requires the knowledge of complete and well-annotated plant genomes, as well as microbial genomes. Metabolomics offers a powerful tool to detect and quantify small molecules and molecular changes at the plant–bacteria interface if the necessary requirements with regard to (secondary) metabolite databases are considered. We highlight data integration and complementarity which should help to widen our understanding of the interactions among individual players of the plant holobiont in the future.

## Background and Experiment Design

### The Relationship of Plants and Their Microbiomes

The relevance of the plant holobiont, inclusive of a myriad of beneficial, mutualistic, and pathogenic microorganisms, has been widely recognized (Sanchez-Canizares et al., 2017). Regarding the vast number of potential and actual combinations of plant species and microbial taxa, it is likely that we are far from understanding their multitrophic interactions and metabolic interdependencies.

Plant microbiomes are often separated into aboveground and belowground constituent parts. Leaves, stem, and reproductive organs form the phyllosphere/phyllobiome, while roots and the small volume of associated soil represent the rhizosphere/rhizobiome (Bringel and Couee, 2015; Oburger and Schmidt, 2016). Bacterial (and to a lesser extent archaeal) populations within these compartments can be subdivided into epiphytes that colonize the exterior surface of plant tissue and endophytes that penetrate the outermost plant cell layer (epidermis) and colonize interior parts intercellularly and intracellularly. Although often investigated separately, individual plant compartments may not represent entities that restrict the transition from rhizosphere to leaf *vice versa* (Bai et al., 2015) but could be considered as systems with restricted accessibility for microorganisms. Classifying plant-associated fungi according to the site of colonization is straightforward. pathogenic fungi are generally subdivided into ectomycorrhiza that develop a mantle around root tips and penetrate into intercellular root spaces and endomycorrhiza that form arbuscules and colonize intracellularly (Venturini and Delledonne, 2015). however, mycorrhiza bridging those two subtypes have been observed (Yu et al., 2001; Navarro-Rodenas et al., 2012) rendering the spatial definition of colonization less definite. recently, a high diversity of ectomycorrizal operational taxonomic units (Otus) was observed for a single root system including a clear spatial structuring with regard to root age (Thoen et al., 2019). Non-mycorrizhal, the rice plant pathogen *Magnaporthe oryzae* establish epiphytic and endophytic associations, depending on the stage of the life/infection cycle (Wilson and Talbot, 2009). Owing to this variety of spatial interactions among plants and microorganisms, researchers have to be aware of microbial colonization characteristics and potential transitions between plant exterior and interior, and even organs.

### Experimental Design Including Sampling and Replication

Planning of field and greenhouse experiments should account for potential spatial effects by grouping experimental units into blocks (e.g., randomized block design) (Clewer and Scarisbrick, 2001). The inherent variability of biological materials, such as root and leaves, necessitates an adequate investment in replication (Prosser, 2010; Lennon, 2011). Leaf- and root-associated microbial populations have been shown to vary in abundance and community structure according to plant development stage (leaves, Copeland et al., 2015; roots, Wagner et al., 2016; leaves and roots, Edwards et al., 2018) rendering a sufficient number of replicates a prerequisite for sound statistical interpretation of sequencing data. In addition, stochastic effects, such as the timing of arrival of species, may have an effect on species distribution on roots and potentially also leaves (Kennedy et al., 2009). We recommend to take at least five replicate samples per plant organ or sample type to compensate for this inherent variability. When root-associated microbiota are to be investigated, we strongly recommend to include bulk soil samples (i.e., soil in distance ≥ 2 cm to roots) (Kuzyakov and Razavi, 2019) and treat these accordingly to obtain information on the reference microbial communities (i.e., the microbial seed bank) from which the root microbiome has been most likely acquired. Moreover, the high variability in microbial colonization density and community structure among plant organs/tissues (e.g., roots, ectomycorrhizal root tips) of individual plants should be considered when young tissues are the object of comparative community analysis (Richter-Heitmann et al., 2016). Nevertheless, researchers should be aware that even a high number of replicates does not necessarily protect from confounding issues through unnoticed differences between samples and controls (Quinn and Keough, 2002) and from stochastic effects.

Sampling of plant material should be performed at the site of plant growth (e.g., field, greenhouse) to prevent changing environmental conditions that impact microbial community composition associated with plant organs (e.g., phylloplane-colonizing bacteria). Consequently, samples should be snap frozen immediately or stored in commercial stabilization solutions (e.g., RNA*later*, LifeGuard), depending on the downstream application and the accessibility of liquid nitrogen or dry ice in the field. Sample preparation steps often include washing with solutes. These reagents should be sterilized and sequenced separately to obtain information about potential contaminations that might affect microbiome analyses and prevent the detection of low-abundance community members (Quinn and Keough, 2002; Laurence et al., 2014; Salter et al., 2014). For example, rinsing roots with tap water and analyzing samples *via* metaproteogenomics (Knief et al., 2012) may potentially cause the risk of adding artefacts/contaminations to data.

Researchers aiming to address research questions through the application of sequencing techniques should be aware of potential artefacts provoked by sampling and treatment of plant material and associated microbiomes. For example, studies addressing root–microbe interactions often try to separate soil–#root interfaces into the rhizosphere (soil attached to roots), the rhizoplane (actual root surface), and endosphere (root interior). While washing-off soil from the root and obtaining a “rhizosphere” sample is rather straightforward (although not all soil will be removed by washing), the differentiation of rhizoplane- and endosphere-associated microbial populations is not trivial (Richter-Heitmann et al., 2016). Here, additional washing and shaking of roots may only decrease the number of cells attached to the rhizoplane. Consequently, the studied rhizoplane sample will not cover the full diversity and abundance of cells while the endosphere sample will be “contaminated” with remaining soil particles and cells that were not washed off from the rhizoplane. Instead, sonication may help to reduce almost all rhizoplane-associated cells (Bulgarelli et al., 2012; Lundberg et al., 2012) but a disruption of the outer cells close to the rhizodermis may also lead to significant loss of endophyte diversity and abundance as analyzed by downstream sequencing. Surface treatments with sterilizing agents (e.g., sodium hypochlorite) have been evaluated to yield “clean” rhizoplanes while allowing for a sequencing-based investigation of microbial populations. Although downstream complications through penetration of the sterilizing agent into the root/leaf interior cannot be excluded, this treatment represents the method of choice if an endosphere compartment should be investigated upon its microbiome (Reinhold-Hurek et al., 2015; Richter-Heitmann et al., 2016). To our knowledge, studies addressing these questions of separating phyllosphere compartments are missing. Thus, it is of utmost importance to perform rigorous testing of potential separation strategies *via* microscopic observation and media plating of treated specimens to assess the nature/composition of samples that should be investigated by downstream sequencing.

### Common Sources of DNA Contaminations

For obtaining transcriptomic and genomic data sets of plant-associated microbes, it is necessary to set up strategies for reducing non-microbiome DNA to a minimum during experiments as well as *in silico*. Such DNA can originate from different sources. Researchers planning to extract microbial nucleic acids from plant material should be aware that milling and physico-chemical lysis will lead to the co-extraction of chloroplast and mitochondrial DNA (Lutz et al., 2011). As mentioned earlier, samples from plant roots can be highly contaminated due to the challenge in removing the rhizosphere. Human DNA can be also a source of contamination (Kryukov and Imanishi, 2016) when introduced during DNA preparation of the samples. Furthermore, relic DNA can potentially obscure estimates of soil microbial diversity (Carini et al., 2016) which could also impact the analysis of root samples (rhizosphere soil) and other plant tissue.

### Recent and New Approaches to Study Plant–Microbe Interactions

The recent advent in high-throughput sequencing in combination with an array of “omics” techniques allows researchers to identify microbiome structure and dynamics along with host interactions on an unprecedented level. Modern sequencing techniques provide in-depth information on the identity and relative abundance of the microbial partners of plants. Because sequences are generated directly from the environmental sample, the cultivation of microbial isolates is not necessary (Epstein, 2013; Hug et al., 2016). However, the freedom gained through sequencing technology can result in a deluge of data which must be countered by selecting an experimental design and sequencing methodology appropriate to the scientific question being asked. A thorough understanding of the types of expected biases and errors should be considered carefully when choosing a particular sequencing method.

High-throughput sequencing of marker gene amplicons is typically used to elucidate the composition, organization, and spatial distribution of microbial communities in the environment and is increasingly used in plant microbiome studies (Knief, 2014). The advantage of amplicon sequencing is that it can be extremely specific, targeting single groups of microbes (e.g., Bacteria, Archaea) or even functional genes (DsrA, AmoA, etc.) (Herbold et al., 2015). The high specificity of amplicon sequencing allows it to be used to positively identify even rare organisms; however, the sensitive nature also renders amplicon sequencing prone to contamination (Glassing et al., 2016). Therefore, it is essential to include positive (known mock communities) and negative controls (reagent and extraction blanks) for any experiment that relies heavily on amplicon sequencing.

Shotgun metagenomics is less sensitive than amplicon sequencing in being able to verify the presence of rare organisms; however, the abundances measured are less biased (Poretsky et al., 2014), and the data can be “binned” into draft genome sequences. These enable one to tie taxonomic identity to functions important to plants, such as nitrogen fixation, or to determine whether symbionts might have the ability to “communicate” with plants *via* secretion systems or effectors (Eichinger et al., 2016).

Metagenomic approaches can be complemented by other high-throughput molecular techniques, such as transcriptomics, proteomics, and metabolomics. Metatranscriptomics are well established in human microbiome research (Bashiardes et al., 2016) and can serve as a blueprint for application in plant microbiome research. Best practices for RNA-seq data analysis have been reviewed recently (Conesa et al., 2016). Metaproteomic data can not only be used as evidence for protein expression and quantification but also to refine gene models (Nesvizhskii, 2014), identify posttranslational modifications, frameshifts, and offer insights into entire microbial communities in plants (Butterfield et al., 2016). Studying plant metabolomes gives information on primary and secondary plant metabolites that may interact with the microbiome within the host (plant solute transport), as well as on the exterior surfaces (phylloplane, rhizoplane) through secretion as exudates (van Dam and Bouwmeester, 2016).

Bioinformatic analysis has substantially contributed to our understanding of microbial roles and their interaction with plants (Marasco et al., 2012; Koberl et al., 2013; Spence et al., 2014; Cha et al., 2016). For example, the identification of *Pseudomonas* spp. as the cause of sugar beet affection in soil suppressive to *Rhizoctonia solani* was initially based on metagenomic data analysis (Mendes et al., 2011). However, often, it is not trivial to test computational predictions under controlled conditions in the lab or in the field. Recent work toward engineered plant microbiomes includes computational modeling (Scheuring and Yu, 2012) and synthetic community experiments combined with multi-omics (Vorholt et al., 2017).

### Aim of This Review

All approaches introduced, so far, require specific bioinformatics methods and tools for data reduction, analysis, and interpretation. Here, we give researchers a guideline for the computational aspects of planning and performing studies on plant–microbe interactions. We discuss quality of public genome data, software pipelines to analyze amplicon and metagenomic sequencing data, and present workflows of data analysis for both approaches. Data integration of additional “omics” techniques will be addressed to promote a much-needed multidisciplinary research that could shed light into the interlinked complexity of plant–microbiome interactions and their dynamics.

## Microbiome Sequences Inside Plant Genome Assemblies

The DNA extracted from plants for plant sequencing projects can, depending on plant sterilization, sampling, and DNA extraction, contain other eukaryotic, microbial, and viral DNA. Although unintended, plant genome sequencing projects may include DNA from members of their microbial communities. This phenomenon is well known for animal genomes. For example, the genome sequencing project of *Hydra magnipapillata* produced an almost complete genome of a stably associated novel bacterium (Chapman et al., 2010). More recently, re-analysis of the tardigrade genome assembly for *Hypsibius dujardini* demonstrated that horizontal gene transfer (HGT) accounts for at most 1% to 2% of genes in the genome and that the original proposal that one sixth of tardigrade genes originate from functional HGT events was an artifact of undetected contamination (Koutsovoulos et al., 2016).

However, computational mining for microbial contigs in plant genome drafts or detection of contaminations in such assemblies has been limited. Intrinsic information, such as k-mer frequencies and sequencing coverage, indicates regions of unexpected characteristics, which might consist of foreign DNA, HGT or repeats (Delmont and Eren, 2016; Mapleson et al., 2017). Database searches identify regions of unexpected similarity between unrelated genomes, which are candidates for HGT or contamination (Delmont and Eren, 2016; Borner and Burmester, 2017). Assembly evaluation tools, like REAPR (Hunt et al., 2013), assist in identifying potential mis-assemblies from contradictions between paired-end read pair alignments to contigs. However, none of these approaches alone allows reasonably specific detection of contamination in genomes assemblies. Guided by computational predictions, re-assembly and/or resequencing of questionable genomic regions is, therefore, the only reliable strategy to correctly assemble plant genomes.

To get an impression of the current status of possible microbial contaminations on plant genomes, we adapted the database-centric approach used earlier the for draft assembly of domestic cow, *Bos taurus* (Merchant et al., 2014). All latest assembly versions of plant genomes in NCBI Genbank (Benson et al., 2017) were split into chunks of 10 kb, overlapping each other by 5 kb. We screened with the Kraken2 software (Wood and Salzberg, 2014) using a confidence score of 0.5 for microbial and viral contamination (**Table S1**).

**Figure 1:**
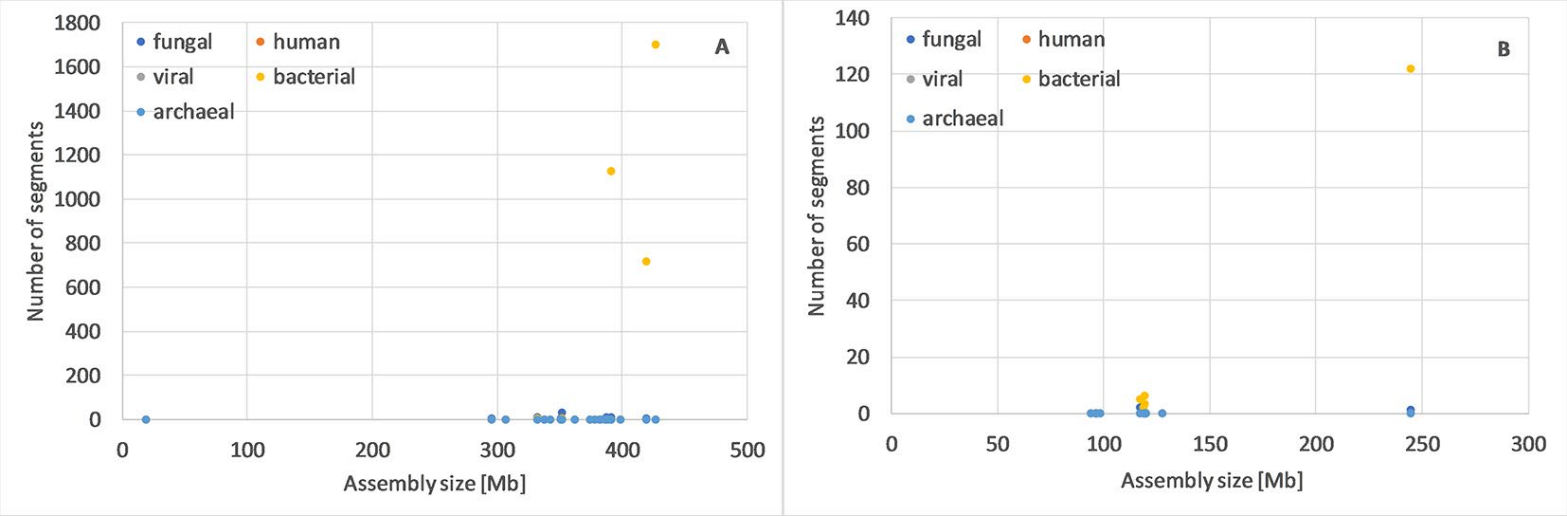
Number of potential archaeal, bacterial, fungal, human and viral of min. 10kb segments in genome assemblies of *Oryza sativa* **(A)** and Arabidopsis thaliana **(B)**. For these two species, it is most clear that one or few assemblies have many more potentially foreign segments than others, independent from the number of contigs or genome size (complete data in **Table S1**).

For *Oryza sativa* and *Arabidopsis thaliana* (**Figure 1**), as well as several other species (**Table S1**), we observed that one or more genome assemblies had more archaeal, bacterial, fungal, human, and/or viral segments than typical. Such assemblies would be reasonable targets for assembly evaluation, reassembly or even resequencing.

## Community-Based Analysis by Amplicon Sequencing

### Primers Commonly Used for Amplicon Generation

Phylogenetic analyses of archaeal and bacterial communities within the plant rhizosphere rely mainly on the gene encoding the small RNA subunit of the ribosome, the 16S rRNA gene. To date, the most widely used next-generation sequencing (NGS) technology for targeted gene analysis is Illumina MiSeq. This technique requires small fragments (250–600 bp) and can generate high-sequencing coverage at low costs (Kozich et al., 2013). For this reason, most studies target the so-called hypervariable regions, e.g., V3, V4, and V5 in the 16S rRNA gene, as they provide sufficient classification accuracy (Liu et al., 2007; Claesson et al., 2010). The variable region V4, which is targeted by the primer pair 515F/806R, was recommended by the Earth Microbiome Project (Gilbert et al., 2014) and has been used in several studies to assess microbial communities in soil (Bates et al., 2011) and in the plant-rhizosphere (Breidenbach et al., 2015). Due to limited coverage of the originally designed primer pair 515F/806R (Parada et al., 2016), primers were updated and recently renamed to “515F (Parada)” (Parada et al., 2016) (and “806R (Apprill)” (Apprill et al., 2015), which provide the most comprehensive coverage of the commonly used 16S rRNA primer pairs (Eloe-Fadrosh et al., 2016). There are certainly other 16S primers available that are used in plant microbiome studies, as shown in **Table 1**, but some of them have limited coverage. Profiling of fungi is performed with the internal transcribed spacer (ITS) between the small (18S) and the large (28S) subunit ribosomal rRNA gene and most commonly used primers are ITS1f and ITS2 (Gilbert et al., 2014; Tedersoo and Lindahl, 2016). The ITS2 region has recently been suggested as a better-suited target to extend the coverage of the fungal kingdom, using new and improved primers (Nilsson et al., 2019).

**Table 1:** Coverage of bacteria, archaea, and chloroplasts by 16S rRNA amplicon sequences amplified with primer pairs used in plant-associated microbiome studies.

Publication	PMID or doi	Fw_primer_name	Fw_primer_sequence	Rv_primer_name	Rv_primer_sequence	Bacteria_coverage	Archaea_coverage	Chloroplast_coverage
(Caporaso et al., 2011)	20534432	515f	5’-GTGCCAGCMGCCGCGGTAA	806r	5’-GGACTACHVGGGTWTCTAAT	88	56.6	74.5
(Apprill et al., 2015; Parada et al., 2016)	26271760, doi.org/10.3354/ame01753	515f (Parada)	5’-GTGYCAGCMGCCGCGGTAA	806rB (Apprill)	5’-GGACTACNVGGGTWTCTAAT	88.7	89.5	74.6
(Lundberg et al., 2012)	22859206	1114F	5’-GCAACGAGCGCAACCC	1392R	5’-ACGGGCGGTGTGTRC	68.6	0	74.9
(Lundberg et al., 2012)	22859206	926F(mod)	5’-AAACTYAAAKGAATTGACGG	1392R	5’-ACGGGCGGTGTGTRC	80.5	1.1	81.9
(Lundberg et al., 2012)	22859206	804F	5’-ATTAGATACCCDRGTAGT	1392R	5’-ACGGGCGGTGTGTRC	74	60.6	3.9
(Bodenhausen et al., 2013)	23457551	799F	5’-AACMGGATTAGATACCCKG	1193R	5’-ACGTCATCCCCACCTTCC	60	0	0
(Beckers et al., 2016)	27242686	799F	5’-AACMGGATTAGATACCCKG	1391R	5’-GACGGGCGGTGWGTRCA	74.5	56.2	0.8
(Klindworth et al., 2013)	22933715	341F	5’-CCTACGGGAGGCAGCAG	785R	5’-GACTACHVGGGTATCTAATCC	84.5	0.1	61.8

Primer pairs were tested using TestPrime against the SSU 132 SILVA database (0 mismatches, Sequence Collection: Ref).

### Co-Amplification of Plastids and Mitochondrial DNA

Universal 16S primers come with a limitation: they can also amplify plastid and mitochondrial DNA. This issue is highly relevant for plant associated microbiome studies, considering the abundance of organelles in a plant derived DNA sample. Fortunately, the undesired amplification of organellar DNA may be partially overcome through primer choice. The first primer (799F) designed to diminish the amplification of chloroplast sequences was introduced by Chelius and Triplett 15 years ago (Chelius and Triplett, 2001). The 799F primer, combined with the 1391R, can reach up to 74.5% and 56.2% coverage of all bacterial and archaeal 16S rRNA sequences, respectively, while amplifying only 0.8% of sequences classified as chloroplast in the SILVA database (**Table 1**). This pair has been also experimentally tested in a recent study of plant-associated bacterial communities (Beckers et al., 2016). In addition to primer optimization, a promising means of limiting the unwanted amplification of chloroplast and mitochondria sequences is the application of PCR clamps. These synthetic oligomers have been reported to physically block the amplification of plant host DNA while increasing the number of microbial 16S rRNA sequences (Lundberg et al., 2013; Blaustein et al., 2017). A useful resource of 16S individual primers used for detection of plant associated prokaryotes has been compiled recently [**Supplementary Table 2** of (Reinhold-Hurek et al., 2015)]. To also provide the theoretical coverage of widely used primer pairs, we have performed an *in silico* analysis using TestPrime against the SSU 132 SILVA database (Klindworth et al., 2013) without mismatches and sequence collection “Ref” (**Table 1**).

In addition to 16S rRNA and fungal ITS, amplicon sequencing studies consider functional genes as phylogenetic marker which are normally enzymes that are involved in major biogeochemical processes in soils and the plant rhizosphere. Of particular importance are *pmoA* for methanotrophs (Suddaby and Sourbeer, 1990), *amoA* for ammonia oxidizers (Pester et al., 2012), *nxrB* for nitrite oxidizers (Pester et al., 2014), *nifH* for diazotrophs (Collavino et al., 2014; Angel et al., 2018), *mcrA* for methanogens (Zeleke et al., 2013), and *dsrB* for sulfite/sulfate reducers (Zeleke et al., 2013; Pelikan et al., 2016; Jochum et al., 2017; Liu and Conrad, 2017; Vigneron et al., 2018). A quite comprehensive overview of functional genes is provided at the Fungene database (Fish et al., 2013).

### Amplicon Sequencing Protocols

Once a proper primer pair is selected, compatibility with the respective sequencing platform (e.g., Illumina Miseq) has to be ensured. For example, Illumina’s adaptors are added to the primer sequence and short barcodes in primer sequences enable sequencing of many samples in parallel. This can be achieved either by a single PCR step with primers that already incorporate the barcode and the adapter (Caporaso et al., 2011) or by a workflow in which the template is first amplified, and barcodes are later added in a second-step PCR before the ligation of the adaptors (Herbold et al., 2015). In the latter approach, the same barcode can be combined with different primers.

Alternative strategies for sequencing amplicons also exist. For instance, PacBio sequencing, which can be sufficient for sequencing the entire 16S gene or in general fragments up to 30 kb (Armanhi et al., 2016; Singer et al., 2016). All sequence data are a useful resource for the scientific community, and archiving such data ensures reproducibility in research. Hence, it is required that all sequences are submitted to the INSDC Sequence Read Archive (SRA) (Cochrane et al., 2016).

### Processing of Amplicon Sequencing Data

To obtain biologically meaningful results from NGS data, it is necessary to thoroughly process sequences to denoise reads into amplicon sequence variants (ASVs) and/or group them into reliable OTUs. OTUs were originally proposed as a pragmatic alternative to species-level classification to aid in quantitative ecological comparisons (Sokal & Sneath: *Principles of Numerical Taxonomy*, San Francisco: W.H. Freeman, 1963) and are a common feature of modern microbial ecology. A handful of tools exist for forming OTUs, e.g., Mothur (Schloss et al., 2009), QIIME (Caporaso et al., 2010), and UPARSE (Edgar, 2013). All three pipelines contain similar processing steps, e.g., quality and length filtering of sequencing reads and OTU generation and classification of microbial 16S rRNA amplicons. For processing of ITS amplicons, PIPITS (Gweon et al., 2015) represents a collection of commands that require software, such as VSEARCH (Rognes et al., 2016), an open-source software analogue to USEARCH (Edgar and Flyvbjerg, 2015). For a more in-depth review of ITS amplicon sequencing we refer the reader to (Nilsson et al., 2019).

An evaluation of the available tools and parameters for read processing is beyond the scope of this review but can be found elsewhere (e.g., Kopylova et al., 2016). Here, we attempt to provide recommendations for optimal sequence processing into OTUS. (I) Employ paired-end sequencing and merge reads into contigs. Due to a drop in quality in Illumina sequencing reads from 5’ to 3’, error may accumulate and give rise to false diversity. Paired-end reads may allow for a correction of these sequencing errors in the overlapping regions (Schirmer et al., 2015). (II) Remove singletons. The removal of singletons was recommended to further improve data quality, e.g., to remove spurious OTUs (Zhou et al., 2011). (III) Pre-cluster. Pre-clustering prior to OTU clustering simplifies the data, reduces memory requirements and was shown to help in denoising (Huse et al., 2010). (IV) Remove chimeras. PCR and sequencing chimeras are a common problem, and their removal is essential (Quince et al., 2011). Typically used pipelines include multiple chimera detection and removal strategies, which can generally be done with and without reference databases. (V) Contaminant removal. One critical aspect of amplicon data analysis is to track down and remove any contamination inherent to the experimental setup. Therefore, it is obligatory that negative controls are included in the study design. Common sources of contamination are the PCR reagents used in the preparation of the sequencing libraries and barcode crosstalk. Decontam is a statistical framework for detecting contaminants that is available as an R package (Davis et al., 2018) (VI) Normalize library size. The total reads per sample (sequencing library) can vary by orders of magnitudes within a single sequencing run. Therefore, OTU tables have to be normalized for a range of statistical applications. To date, it is standard to rarefy the data, e.g., randomly subsample the reads at the smallest library size (Schloss et al., 2009; Caporaso et al., 2010, REF). However, this method does not acknowledge the zero-inflated data structure and potentially excludes useful information. Other suitable normalization strategies are based on a negative binominal distribution model (McMurdie and Holmes, 2014; Weiss et al., 2017) or by rarefying multiple times (see supplement in McMurdie and Holmes, 2014). (VI) Consider alternatives to OTUS.

The commonly used 97% identity level for *de novo* clustering has been defined to compensate for sequencing errors but may fail to capture sequence diversity of ecological importance in some cases. Fine- or strain-resolved analysis of amplicon data could instead be based on 100% sequence resolution, so-called amplicon sequence variants (Callahan et al., 2017), which requires specific error–correction algorithms and is an emerging field in bioinformatics for amplicon analysis. Currently utilized methods include SWARM(v2) (Mahe et al., 2014; Mahe et al., 2015), minimum entropy decomposition (MED) (Eren et al., 2015b), and DADA2 (Callahan et al., 2016). These methods differ in the algorithm used to identify ASVs, but all produce a set of sequences and an occurrence table that is analogous to OTUs defined at 100% identity, but free from sequencing error. This approach can differentiate between distinct ecologically relevant taxa that would be otherwise be overclustered into a common OTU at a 97% identity threshold (Eren et al., 2015b).

Data normalization as well as compositional nature of relative abundance data dictate what statistical methods should be applied downstream (Gloor et al., 2017). Multivariate analysis includes methods to explore variance, interpret relationships in the light of constraint variables and even define discriminant functions (Paliy and Shankar, 2016). Detailed overview of the statistical methods for the analysis of amplicon data has been described previously (Hugerth and Andersson, 2017).

### Databases and Methods for Sequence Classification

To facilitate data interpretation, OTUs and/or ASVs need to be classified into recognized taxonomic groups. A widely applied software for this purpose is the Ribosomal Database Project (RDP) classifier (Wang et al., 2007), which uses k-mer fragments of an OTU sequence to identify the closest matching organism in a reference database. This classifier is implemented in the mothur and QIIME software packages and can be used for classification of NGS amplicons generated from 16S rRNA genes (e.g., Breidenbach et al., 2015) as well as from ITS genes (Gweon et al., 2015). One of the most recent and supposedly faster k-mer based database search tool is IDTAXA (Murali et al., 2018). Useful alternatives to k-mer based search tools are least common ancestor-based methods, such as SINA (Pruesse et al., 2012) or CREST (Lanzen et al., 2012) and tools based on phylogenetic placement, such as the evolutionary placement algorithm (EPA) (Berger et al., 2011) and pplacer (Matsen et al., 2010). These tree reconciliation methods generally have a higher classification accuracy at a higher phylogenetic level and are, therefore, suitable for detection of novel taxa (Matsen et al., 2010; ; Lanzen et al., 2012; Pelikan et al., 2016; Angel et al., 2018).

For 16S rRNA gene classification, the databases Greengenes (DeSantis et al., 2006), Silva (Quast et al., 2013), and RDP (Cole et al., 2014) are most widely used. Databases for ITS sequence classification are UNITE (Abarenkov et al., 2010) and WARCUP (Deshpande et al., 2016). As chloroplasts are likely co-amplified with the plant microbiome (Lundberg et al., 2012), sequences that are classified as chloroplasts by any of the above-mentioned tools should therefore be removed from the sequence data set.

### Analysis of Amplicon Sequencing Data (Including Online Resources)

Once OTUs are generated, their abundance matrix has to be analyzed. A good overview for microbial ecologists about the available statistical analysis methods and their usability was compiled earlier (Ramette, 2007), which later resulted in a useful online resource called GUSTAME (Buttigieg and Ramette, 2014). Tools for statistical analyses mainly rely on the R software (R Core Team, 2017) and specifically on the vegan software package (Oksanen et al., 2017). Tools for non-expert users can be divided into interactive R-based online resources, such as phyloseq shiny (McMurdie and Holmes, 2015) and Calypso (Zakrzewski et al., 2017), easy-to-use and well-documented software packages for commandline R, such as phyloseq (McMurdie and Holmes, 2013) and Rhea (Lagkouvardos et al., 2017); and standalone programs, such as mothur and QIIME.

### Limitations

Despite its popularity in characterization of microbial communities, known biases of amplicon sequencing should not be neglected. Universal primers amplify genes from different taxonomic lineages with different efficiency (Hong et al., 2009; Schloss et al., 2011; Mao et al., 2012). 16S genes with long introns might be missed by typical PCR design due to their length (Salman et al., 2012; Brown et al., 2015). Different numbers of rRNA gene clusters per genome have direct impact in estimating the relative abundance of individual bacterial taxa (Vetrovsky and Baldrian, 2013). Furthermore, unless specific functional genes are being used, where there is a congruence between the function and phylogeny, amplicon sequencing is not ideal for inferring community function, although there are available methods (Langille et al., 2013).

## Shotgun Metagenomic Approaches

Whole genome sequencing utilizes sequence information from the entire genome, which represents different levels of conservation. Compared to amplicon sequencing this provides better phylogenetic resolution and enables function prediction. However, to leverage the richness of metagenomic data sets to answer targeted scientific questions, it is first important to consider how much sequencing data is necessary. Unfortunately, this is not a straightforward task. Plant-associated communities tend to be complex, with a high level of strain diversity that can result in lower coverage of specific genomes and poorer assembly (Sczyrba et al., 2017). Strategies to estimate how much sequencing is necessary to recover information for a target genome require existing 16S rDNA amplicon data (Tamames et al., 2012; Ni et al., 2013) and/or a preliminary metagenomic data set (Rodriguez et al., 2018). Although very small metagenomic data sets may be suitable for assessing taxonomic richness (Kwak and Park, 2018), it should also be kept in mind that the sequencing depth will have a direct impact on what scientific conclusions can be drawn (Sczyrba et al., 2017; Zaheer et al., 2018). Therefore, it is important to carefully evaluate the reason a metagenomic data set will be generated and determine the necessary sequencing depth according to the type of analysis that will be conducted.

Four techniques are typical for the computational analysis of shotgun metagenomes: 1) taxonomic binning, 2) taxonomic profiling, 3) target–gene reassembly, and 4) genome binning.

### Taxonomic Profiling and Binning

Taxonomic profilers and taxonomic binners use existing databases to assign unassembled sequence data into known taxonomic groups. Numerous methods aimed at producing taxonomic profiles and taxonomic bins have been developed (**Table S2**). For extensive performance-based reviews, please see Lindgreen et al. (2016) and Sczyrba et al. (2017). Taxonomic profilers and taxonomic binners use existing databases to assign unassembled sequence data into known taxonomic groups. Taxonomic profilers produce tables of abundances per taxa, either based on presence/absence or relative abundance of taxonomic groups, similar to taxonomic marker-based amplicon analyses. Profiling tools map read data against a database of single-copy gene markers (e.g., MetaPhlAn2; Truong et al., 2015), match k-mers to genomic databases (e.g., CLARK; Ounit et al., 2015) or match gene composition against the gene composition found in genomic databases (e.g., Taxy-pro; Klingenberg et al., 2013). These procedures assign a taxonomic lineage to each read, tabulating relative abundance profiles of microorganisms across a set of metagenomic samples. Methods relying on databases of single-copy gene markers use a small fraction of the total read data, since single-copy markers represent a small proportion of an organism’s total DNA. Methods that instead use a database of whole genomes assign many more reads to taxonomic groups resulting in greater recall of rare taxa, however, do not ensure that greater precision or accuracy is achieved for the calculated relative abundances (Sczyrba et al., 2017). Taxonomic binners work in a similar fashion as taxonomic profilers, however, aim to collect reads or contigs into taxonomic groups rather than produce a taxonomic profile of presence/absence or abundance. Reads assigned to taxonomic groups can subsequently be mined for function or assembled independently from reads assigned to other taxonomic groups (Cleary et al., 2015). Taxonomic bins can be specified at any taxonomic level (species, genus, order, etc.), however, tend to perform poorly at the genus and species level. These methods also suffer from the assignment of data into mixed small bins, which should be discarded (Sczyrba et al., 2017).

### Target Gene Assembly

Another popular method for characterizing the taxonomic composition of a metagenome is the reconstruction of partial to full-length rDNA sequences directly from raw metagenomic data sets (Miller, 2013; Gruber-Vodicka et al., 2019). This technique differs from taxonomic profiling. Although both techniques rely initially on mapping raw data to a database, the aim of target-gene assembly is to reconstruct full-length genes that can be directly used in downstream applications, such as detailed phylogenetic analysis, the identification of novel taxa and precise taxonomic classification (Soergel et al., 2012). Furthermore, this procedure is free from the PCR bias inherent to amplicon-based techniques and thus may better estimate ribosomal gene abundances in the environment. The procedures used to reconstruct full-length rDNA sequences have also been adapted to allow the reconstruction of protein-coding functional genes (Orellana et al., 2017). These targets include functional process marker genes such as ammonia monooxygenase (Rotthauwe et al., 1997), dissimilatory (bi)sulfite reductase (Wagner et al., 2005) and dinitrogenase reductase (Widmer et al., 1999), which can act as both taxonomic and process markers.

### Binning Genomes From Metagenomic Data Sets

**Figure 2:**
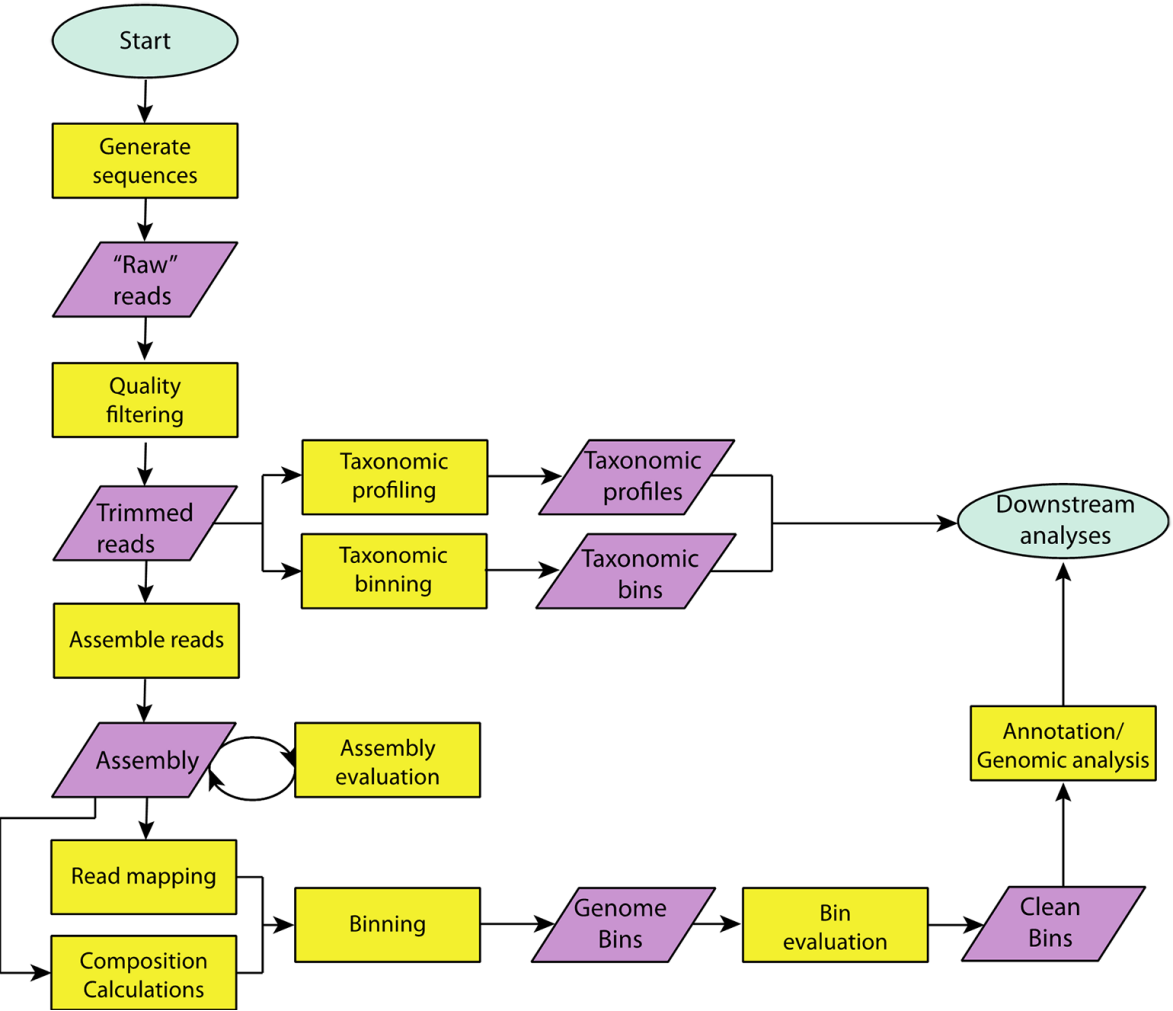
A flowchart outlining steps taken in a typical metagenome analysis. Specific tools, which can be used to carry out each step can be found in **Supplemental Tables S2 and S3**.

Genomic bins, known as metagenome-assembled genomes (MAGs) obtained from metagenomic data sets have revolutionized our understanding of the tree-of-life (Hug et al., 2016). The workflow for generating MAGs is shown in **Figure 2**. Here, we outline the overall approach and provide lists of specific tools available in **Table S3**.

Robust binning is dependent on a reliable assembly. To generate a high-quality assembly, raw sequencing reads are first trimmed and filtered to improve quality. Reads with overall low quality are usually discarded, while the remaining reads are trimmed to remove low-quality ends and adaptor sequences. Reads are then assembled using programs that have been optimized specifically for complexity and varying genome coverage which is typical in metagenomic data. Systematic evaluation of typical assemblers (Sczyrba et al., 2017) showed that complex data sets result in poorer assemblies. User-defined parameters should be explored and adjusted to obtain the optimum assembly. A useful tool for evaluating assemblies constructed under different parameter settings is MetaQUAST (Mikheenko et al., 2016).

Genomic binning algorithms rely on two different types of information, composition, and coverage, for differentiating genomes. Compositional information, such as GC content or tetranucleotide frequency, has long been exclusively used to separate MAGs from one another. Contigs or scaffolds below some length are usually excluded, as composition-based statistics are weaker, and accuracy of classification quickly declines (Sandberg et al., 2001). To calculate coverage, raw or quality-checked reads are mapped against assemblies using any one of several programs. Binning algorithms measure tetranucleotide frequency patterns contained within scaffolds and, in combination with the information on coverage across samples, classify scaffolds into individual MAGs. A crucial step is then to evaluate the quality of the obtained MAGs. The contamination and completeness level should be measured to characterize the MAG as a single genome or a set of closely related genomes (Parks et al., 2015; Waterhouse et al., 2017). These statistics are important when making arguments regarding absence or co-occurrence of genes (e.g., inferring pathways). Several tools have been developed to evaluate the quality of MAGs (see **Table S3**) and a detailed set of standard guidelines [minimum information about a metagenome-assembled genome(MIMAG)] has been developed by the Genomic Standards Consortium for reporting and evaluating MAGs (Bowers et al., 2017). Once a MAG passes the necessary quality assessment, it can be treated nearly the same as a draft genome from culture. Gene prediction and functional annotation of predicted protein sequences within each bin can be computed using available automated pipelines. Automated tools with predefined workflows, like ATLAS 2.1.4 (https://metagenome-atlas.readthedocs.io/en/latest/) or Anvi’o (Eren et al., 2015a), are useful aids for metagenomic data analysis without requiring extensive bioinformatics skills. Also, the *in situ* replication rate of MAGs can be estimated using iRep (Brown et al., 2016), giving insight into which organisms may have been replicating at the time of sampling.

### Publicly Available Plant-Associated Metagenomes

Many studies may benefit from a comparison of newly generated data to existing data and several databases host publicly available data to enable such comparisons. The availability of plant-associated metagenomic data from three resources is summarized in **Table 2**. It should be noted that these data may be redundant between the three resources. They are not necessarily mutually exclusive, and a single data set can be hosted by one or more resource.

**Table 2:** Plant-associated metagenomic data set availability in publicly available databases.

Search term	ENA- SRA metagenomes	MG-Rast metagenomes	IMG metagenomes
Rhizoplane	0	0	197
Rhizosphere	1450	137	78
Phyllosphere	33	25	33
Endophyte	552	0	0
Endosphere	0	0	10
Nodule	0	0	3
Roots	112	12	1
Rice Paddy	77	23	0
Root-associated fungus	13	0	0
Shoot	12	0	0
Leaf	10	0	0
Pollen	2	0	0
Moss	748	1	6
“Plant” (unspecified)	469	0	0

Counts refer to total number of discreet data sets, including biological and technical replicates.

An extensive resource of publicly available metagenomic data is hosted at the Short Read Archive (SRA) of the International Nucleotide Sequence Database Collaboration (INSDC) (Cochrane et al., 2016). A requirement for deposition into this resource is the inclusion of minimum information about a metagenome sequence (MIMS) (Field et al., 2008) enabling an efficient path to identify and download raw data for comparative analysis. Data deposited in the SRA has been minimally processed, so that it can be processed alongside newly generated data using any chosen pipeline to ensure maximum comparability. A second resource of raw metagenomic data is the MG-Rast server (Meyer et al., 2008). Although not as comprehensive as the SRA, MG-Rast processes raw reads through a standardized pipeline to produce taxonomic and functional profiles and calculate diversity statistics. Users can upload their newly generated data sets and analyze them with the standard pipeline. Pipeline standardization coupled with periodic re-analysis of existing data sets, ensures that newly deposited data can be compared directly to previously deposited data. A third resource for metagenomic data is IMG/M (Markowitz et al., 2014). Instead of raw data, IMG/M primarily hosts assembled data (contigs, scaffolds, MAGs). IMG/M also uses a standardized pipeline to compare the newly deposited data sets to the existing IMG/M database, which includes an extensive collection of complete and draft genomes as well as a metagenome to infer taxonomy and function.

A potential hurdle to a meaningful comparison of newly generated metagenomic data to pre-existing data is the lack of consistently applied ontology in metadata entries. For each of the aforementioned databases, scientists are responsible for uploading data which can result in non-uniform usage of metadata terms. For instance, the separation of samples into rhizoplane and rhizosphere compartments is experimentally difficult (Reinhold-Hurek et al., 2015; Richter-Heitmann et al., 2016) and no data sets found in the SRA or in MG-Rast possess a “rhizoplane” label (**Table 2**). Researchers interested in comparative metagenomics of the rhizoplane would need to use additional metadata or contact the depositor of metagenomic data to distinguish between rhizoplane and rhizosphere data. In addition, many sequencing projects are funded using public funds, and it is recognized that such data should be available to the public as soon as possible. It is therefore important to note that publicly available data has not necessarily been published. Protocols are in place to reserve publication rights for the “data providers,” or researchers who conduct sample collection and/or experiments that are used to generate sequence data. The Fort Lauderdale (2003) and Toronto (Toronto International Data Release Workshop et al., 2009) agreements provide general guidelines for the use of publicly available data.

## Additional Omics Strategies and Their Integration With Microbiome Data

### Metatranscriptomics

The amount of transcript sequences from the organisms in a microbiome, under a specific condition, is indicative of microbial activity and function. RNA-sequencing (RNA-Seq) is one of the most popular methods used in transcriptome analysis. The whole plant associated microbial communities was first analyzed with metatranscriptomics in *A. thaliana* rhizosphere, at different developmental stages (Chaparro et al., 2014). When sequencing RNA it is important to consider the high abundance of ribosomal RNA (rRNA) molecules in the cell. rRNA can be considerably reduced by using special library preparation kits like TruSeq Ribo-Zero (from Illumina). A crucial step in RNA-seq is the construction of complementary DNA (cDNA) from the RNA template by a reverse transcriptase. For this purpose, protocols have been established, and they can be classified into two categories: stranded and non-stranded (strand information is lost) (Hou et al., 2015). For sequencing plant transcripts, Oligo (dT) primers are used to hybridize to the poly-adenylated tail found on the 3′ ends of most eukaryotic mRNAs. However, for non-eukaryotic organelles, there are no specific tails, and random primers must be used. Therefore, in plant microbiome RNA-seq, it is expected to find sequences from the host RNA. Ideally, RNA sequencing is deep enough to also cover lower expressed transcripts. Recommendations for experimental design and sequence depth are provided by the ENCODE consortium (https://www.encodeproject.org/about/experiment-guidelines). Third-generation sequencing methods, such as PacBio or Nanopore, provide sequence read lengths up to hundreds of kbp (Bronzato Badial et al., 2018; Minio et al., 2019), enabling sequencing of complete transcripts. These technologies still have a high error rate that can be reduced by deep sequencing. In practice, however, these technologies are often not feasible due to high sequencing costs. As a result, most of the metatranscriptomic studies rely on short-reads obtained from Illumina sequencing.

The analysis of short-read metatranscriptome sequences can be addressed in two ways: read-based or assembly-based. Assembly based transcripts can be reference-based (alignment of reads to genome sequences or metagenomic bins) or reference-free (based on metatranscriptomic reads only). Reference-based assemblies have high quality, but only cover those species which genomes could be binned well (unlikely for low abundant and micro-diverse species). Reference-free assemblies suffer from many artefacts (no clear validation method) and from assembly limitations due to homologous gene regions between closely related strains, alternative splice forms, close paralogs, and close homologs (Gongora-Castillo and Buell, 2013).

RNA-seq analysis requires pre-processing of the data, in which rRNA is separated, sequence tails (e.g., long poly-A tail) are removed, and low-quality bases are trimmed. In plant microbiome analysis, the RNA from the host can be separated by mapping the reads to a closely related reference plant genome or transcriptome (if available). In the read-based approach, rRNA and non-rRNA reads are analyzed separately by aligning them to a reference database (e.g., NCBI nonredundant protein database (Coordinators, 2014) for mRNA and SILVA (Quast et al., 2013) for rRNA).

Reference-based assembly methods work in combination with complete genome sequences or high-quality genome bins generated from metagenomic data. In this approach the RNA sequences are mapped to the genomic DNA using intron-aware mapping methods like STAR (Dobin et al., 2013). Reference-free assembly methods rely on *de novo* transcriptome assemblers. Assemblers like Trinity will additionally generate the isoforms of a gene (Grabherr et al., 2011). Taxonomic classification of transcripts is usually based on the lowest common ancestor (LCA) algorithm (e.g., MEGAN; Huson et al., 2007). For comparison between multiple samples, normalization (e.g., based on number of reads) is necessary.

In terms of statistical analysis, transcripts and genes can be quantified with specific methods designed for such purpose, e.g., Kallisto (Bray et al., 2016). For analyzing differentially expressed transcripts or genes (DEG) between samples from different conditions, quantification results are used as input for tools like edgeR (Robinson et al., 2010).

For biological pathway reconstruction, retrieval of gene ontology (GO) terms and gene annotation, a multitude of databases, and associated tools are available. As an example, DEG can be analyzed with blast2GO (Conesa et al., 2005), a software suite which annotates genes with GO terms based on the GO database (Ashburner et al., 2000) and infers biological pathway information based on the Kyoto Encyclopaedia of Genes and Genomes (KEGG) (Kanehisa and Goto, 2000). Significantly overrepresented and underrepresented pathways, functions, or biological processes can be identified based on this information using enrichment analysis of GO terms for DEG. For large scale experimental setups, co-expression networks might be a viable next layer of analysis, as extensively reviewed (Serin et al., 2016).

For a comprehensive annotation of transcriptomes, automatic functional annotation methods like Trinotate are available (Bryant et al., 2017). Trinotate uses a number of different methods for functional annotation, including homology search to known sequence data (BLAST+/SwissProt), protein domain identification (HMMER/PFAM), protein signal peptide, and transmembrane domain prediction (signalP/tmHMM) and leveraging various annotation databases (eggNOG/GO/KEGG databases).

In plant microbial associated studies, metatranscriptomic data were, for instance, used for understanding the rhizosphere microbiome of four crop plants grown in the same soil: wheat (*Triticum aestivum*) oat (*Avena strigosa*), oat mutant (*sad1*), and pea (*Pisum sativum*) (Turner et al., 2013), for assessing bacterial gene expression during *Arabidopsis* development (Lambais et al., 2017). With the decrease of sequencing costs, the use of transcriptomics and metatranscriptomics studies related to plant increased (Levy et al., 2018).

### Metabolomics

Metabolomics studies aim at understanding small molecule metabolites of a biological system under specific conditions. In general, the metabolome consists of primary and secondary metabolites. Compared to other complex biological systems, plants defence mechanisms evolved a high diversity of secondary metabolites (Wink, 2003). Most of them are toxic or repellent to herbivores and microbes. The analysis of metabolomic compounds results in metabolic profiles and fingerprints up to the detection of novel biomarkers, which also can be integrated into microbiome analyses for a more holistic understanding of the plant microbiome.

#### Analytical Technologies Used in Metabolomics

Most frequent technologies used in metabolomics are nuclear magnetic resonance (NMR), gas chromatography-mass spectrometry (GC-MS) and liquid chromatography-mass spectrometry (LC-MS). MS-based techniques detect metabolites with a much higher sensitivity than NMR (Emwas, 2015). However, MS samples require an elaborate preparation, and the detection is limited only to metabolites that are able to ionize into the detectable mass range. The advantages of using NMR stand out for compounds that are difficult to ionize or dissolve or require derivatization for MS (Markley et al., 2017).

Metabolomic methodologies have so far been divided into targeted and untargeted approaches and might merge in the future (Cajka and Fiehn, 2016). The analysis of data obtained from these technologies (NMR and MS) can be divided into pre-processing, annotation, post-processing, and statistical analysis (Spicer et al., 2017). In general, these methods are tailored to the analytical technology. Pre-processing methods are applied to correct the differences in peak shape width and position due to noise, sample differences, or instrument factors (Ren et al., 2015). There is no gold standard pipeline yet for pre-processing of the data. According to the metabolites standard initiative (MSI), for identification, a metabolite must be compared to at least two orthogonal properties of an authentic chemical standard analyzed in the same laboratory with the same analytical techniques as experimental data (Salek et al., 2013). Since most metabolites are not available in the form of chemical standards, they cannot be fully identified. Therefore, MS annotation tools are divided based on different annotation levels (detailed in Schymanski et al., 2014). For NMR, metabolites can be identified by comparing directly with data from online databases (Everett, 2015). This limits the findings to the content of respective databases.

Before statistical analysis, data can be filtered out based on a signal-to-noise ratio selected threshold, or a minimum percent of samples a feature must detect. Normalization is also necessary due to differences in metabolite concentrations in different samples. A current review (Barupal et al., 2018) covers statistical analysis, visualization as well as contextualization of metabolic data from a bioinformatic viewpoint. Lists of freely available tools based on their functionality, and technology used are available in Spicer et al. (2017). The application of multiomics data from genomics, transcriptomics, proteomics, metabolomics, and fluxomics to lipidomics focusing on metabolic modeling in plants have been reviewed recently (Rai et al., 2017).

#### Application of Metabolomics Together With Plant Microbiomes

The vast diversity of soil microbiota interacts with roots of plants, forming a microbial rhizosphere community with intense interactions between plant and microbes. To study such complex interactions, both knowledge about the microbial communities as well as the metabolic constitution of the environment is needed (reviewed in van Dam and Bouwmeester, 2016).

Using a combination of metagenomics and metabolomics, Blaya et al. (2016) could establish a starting point to unravel the complex mechanisms in the suppressive nature of composts to control plant diseases in economically important settings using 16S and ITS for taxonomic analysis together with UHPLC-MS-TOF and additional 13C NMR for the chemical properties of compost and peat. Another multiomics approach using metatranscriptomics and metabolomics could highlight how *Arabidopsis* plants impact soil microbial functions by a changing constitution of root exudates during development of the plant (Chaparro et al., 2013). Plant-microbe interactions play also a vital role in the phyllosphere of plants. Ryffel et al. (2016) applied both NMR and MS methods, investigating epiphytic bacteria on *A. thaliana* leaves and the response of the plant toward epiphytic bacteria and resulting changes in the phylloplane exometabolome. Recently, metabolomics in combination with 16s amplicon sequencing was used to evaluate the potential for metabolic plant–microbial linkages in the rhizosphere of an annual grass in the absence of soil matrix effects (Zhalnina et al., 2018).

#### Bioinformatic Resources and Platforms for Plant Metabolomics

Several online resources are available as well, providing software tools, tutorials, protocols and guidelines on processing, statistical analysis, and visualization of metabolomic data. To this end, platforms like the Metabolic Workbench (Sud et al., 2016), XCMS for MS-based data (Gowda et al., 2014), or MetaboAnalyst (Xia et al., 2015), focusing on biomarker discovery and classification, provide a multitude of resources.

Databases for the annotation of plant genomes and the construction of metabolic models can be obtained from KEGG (Kanehisa et al., 2014) or plant-specific resources as PlantSEED, providing annotation and model-data for 10 plant genomes (Seaver et al., 2014) or Gramene/Plant Reactome as a free and open-source, curated plant pathway database portal (Naithani et al., 2017; Tello-Ruiz et al., 2018). Another vast resource for plant metabolic networks is the Plant Metabolic Network with the PlantCyc database containing 1200 pathways in over 350 plant species as of version 12.0 (Schlapfer et al., 2017).

Overall, complete annotation of plant metabolomes is yet to be achieved, though improvements in non-targeted metabolomics continuously underway (reviewed in Viant et al., 2017).

### Proteomics

Metaproteomics is the study of the proteins in a microbial community from an environmental sample. In contrast to other -omics strategies, metaproteomics provides direct evidence for proteins, post-translational modifications, protein-protein interactions, and protein turnover, reflecting microbial community structure, dynamics, and metabolic activities (Hettich et al., 2013). In general, metaproteomics mostly utilizes methods originating from mass spectrometry (MS)-based proteomics.

#### Experimental Procedures

MS-based proteomics is a powerful analytical technique for large-scale, high-throughput experiments to identify and quantify (characterize) thousands of microbial proteins. In MS-based proteomics we can distinguish between top-down and bottom-up strategies to analyze intact proteins or peptides from artificial proteolytic digestion, respectively. For the purpose of this review, we will focus on the more common bottom-up strategy. In brief, major experimental steps include sample lysis, protein extraction, protein separation, proteolytic digest, peptide fractionation, and MS analysis (Siggins et al., 2012).

#### Computational Proteomics

MS analysis in a large-scale bottom-up experiment readily results in millions of spectra that require automated mass spectral interpretation. Major steps in the computational workflow consist of spectrum pre-processing, peptide identification, quantification (e.g., label-free), protein grouping, and in a metaproteomic context LCA analysis, e.g., UniPept (Mesuere et al., 2018) and Megan (Huson et al., 2007). Peptide identification plays a critical and defining role in metaproteomics to infer most of the constituents of a microbial sample. Among the most popular approaches to assign a peptide sequence to a spectrum are database searching, *de novo* sequencing, e.g., PEAKS (Ma et al., 2003), and spectral library searching, e.g., SpectraST (Lam et al., 2007).

In database searching, a protein sequence database is in silico digested and fragmented to generate theoretical spectra to match against experimental spectra. Most protein sequence databases are built from various omic sources, but at the core use gene predictions from primary genome assemblies. Respectively, the genome quality and its assembly greatly influence the content in reference databases like UniProtKB (Pundir et al., 2017), RefSeq (O’Leary et al., 2016), or Ensembl (Zerbino et al., 2018). In proteomics, one has to balance three aspects of a database, i) complexity to satisfy downstream statistical validation, ii) completeness to identify most constituents, and iii) size to control sensitivity and processing time (Zerbino et al., 2018).

To address those aspects in metaproteomics, various approaches supplement existing reference databases or build custom databases to account for the microbial communities. The proteogenomics field leverages metatranscriptome or metagenome data to build sample specific custom protein sequence databases (Nesvizhskii, 2014). This is especially useful for non-model organisms with no available reference genome database or to identify novel proteins not present in a reference database. Even draft metagenomes provide a sufficient basis to analyze MS data without prior extensive genome annotation (Armengaud et al., 2014). In metatranscriptomics and metagenomics, many short-read assembly algorithms make use of de Brujin graphs as primary data structure to infer primary assemblies. Tang et al. reutilize the graph structure to match MS spectra and construct a more comprehensive database of putative proteins (Tang et al., 2016). A common challenge to metaproteomics and proteogenomics is the loss in sensitivity due to an increase in number of databases or database size (Jagtap et al., 2013). Database size reduction methods include a two-step search method to create a smaller database from a “survey” search and database clustering prior to searching (Marx et al., 2013).

#### Metaproteomics in Plant Microbial-Associated Studies

In plant microbiome studies, metaproteomics were, for instance, used to evaluate bacterial communities in the phyllospheres of tree species in a pristine Atlantic Forest (Lambais et al., 2017), for investigating the response of the plant PGPB *Bacillus amyloliquefaciens* FZB42 to the presence of plant root exudates (Kierul et al., 2015), to determine the differences between the soil protein abundance in plant sugarcane and ratoon sugarcane rhizospheric soils (Lin et al., 2013) and few other studies. Despite the successfully usage, metaproteomics in plant microbiome are limited due to the lower expression of proteins in plant microbial samples and limited information in the databases (Levy et al., 2018).

## Conclusion

The emergence of molecular techniques over the last decades has considerably improved and sped up the analysis of plant-associated microorganisms, e.g., i) deep understanding of *A. thaliana* roots microbiome (Bulgarelli et al., 2012; Lundberg et al., 2012) and ii) identification of key bacterial taxa and genes involved in suppression of a fungal root pathogen (Mendes et al., 2011). However, remaining challenges include: i) understanding the high diversity of plants and their microbiome, ii) assembling useful databases, iii) inherent limitations and error in molecular techniques, iv) moving from model systems to the field. A promising approach to understand reciprocal effects of plants, and their microbiota lies in disassembling plant microbiomes and establishing synthetic microbial communities for reconstitution experiments to study interspecies and intraspecies interactions (Vorholt et al., 2017; Duran et al., 2018). Here, the use of genome-sequenced and fully characterized species would allow for predicting functional interrelations that could be tested in experiments under gnotobiotic conditions.

Due to the high diversity of plants and their sequencing and assembly challenges (Schatz et al., 2012) few plant genomes have been sequenced and well analyzed, while many public plant genome sequences are still represented as a draft. Therefore, experiments conducted in model plants, such as *A. thaliana*, will still help in establishing computational and database resources (Genomes Consortium. Electronic address and Genomes, 2016), from which information can be transferred to other plants (Busby et al., 2017). Furthermore, the 10,000 Plant Genomes Project has the potential to reduce this limit by sequencing representative species from every major clade of embryophytes, green algae, and protists (Cheng et al., 2018). Long-read DNA sequencing techniques (PacBio, Nanopore) are expected to improve the quality of genome and metagenomic-derived sequences and will overcome the binning and assembly limitation in samples with high richness. Despite the differences in the plant microbial community based on plant species, soil, and environment, it is very important to study if core microbiome functions specific to phyllosphere and rhizosphere exist and, if so, to understand interaction mechanisms between core microbes and plants. These insights will be challenged by our understanding of microbiome contributions to plant health and the development of applications in agriculture. With the reduced cost of sequencing a huge amount of omics data from plant microbial community can be expected. However, there is so far no plant microbiome specific database where species or strains could be stored together with the information about plant and environmental condition. The development of such databases needs to be prioritized to enable the functional and ecological interpretation of the upcoming large-scale multi-omics plant microbiome data.

## Author Contributions

RL, HS, and TR wrote the manuscript with contributions of all co-authors. All authors read and approved the final manuscript.

## Funding

We gratefully acknowledge funding from the European Union’s Horizon 2020 research and innovation programme under the Marie Skłodowska-Curie grant agreement No 675657 FlowerPower.

## Conflict of Interest

The authors declare that the research was conducted in the absence of any commercial or financial relationships that could be construed as a potential conflict of interest.
