# Supplementary material for: A Bioinformatics Guide to Plant Microbiome Analysis
Source: Front Plant Sci. 2019 Oct 23;10:1313. doi: 10.3389/fpls.2019.01313 (PMC6819368; doi:10.3389/fpls.2019.01313)
Supplement: Supplementary file 1 [file Table_1.docx]

**Supplementary tables**

Table S1: Number of potential archaeal, bacterial, fungal, human and viral min. 10kb segments in all plant genome assemblies from INSDC, as of January 2019.

|  |  |  |  | Number of segments assigned by Kraken2 | | | |  |
| --- | --- | --- | --- | --- | --- | --- | --- | --- |
| Plant Name | Genome ID | Genome size (MB) | Release date | fungal | human | viral | bacterial | archaeal |
| Actinidia chinensis | GCA_000467755.1_Kiwifruit_v1 | 604,217 | 2013-09-16 | 0 | 0 | 0 | 0 | 0 |
| Actinidia chinensis var. chinensis | GCA_003024255.1_Red5_PS1_1.69.0 | 553,842 | 2018-03-28 | 0 | 0 | 0 | 0 | 0 |
| Aegilops tauschii | GCA_002105435.1_ASM210543v1 | 247,197 | 2017-04-14 | 0 | 0 | 0 | 0 | 0 |
| Aegilops tauschii | GCA_000347335.2_ASM34733v2 | 4310,35 | 2017-10-25 | 0 | 0 | 0 | 1 | 0 |
| Aegilops tauschii subsp. strangulata | GCA_002575655.1_Aet_v4.0 | 4224,92 | 2017-10-17 | 4 | 0 | 0 | 2 | 0 |
| Aegilops tauschii subsp. tauschii | GCA_001957025.1_Aet_MR_1.0 | 4327,32 | 2017-01-19 | 19 | 0 | 0 | 1 | 0 |
| Aethionema arabicum | GCA_000411095.1_VEGI_AA_v_1.0 | 192,488 | 2013-06-12 | 0 | 0 | 0 | 0 | 0 |
| Alnus glutinosa | GCA_003254965.1_ASM325496v1 | 611,874 | 2018-06-19 | 1 | 0 | 0 | 16 | 0 |
| Amaranthus hypochondriacus | GCA_000753965.1_AHP_1.0 | 502,148 | 2014-09-09 | 0 | 0 | 0 | 0 | 0 |
| Amaranthus tuberculatus | GCA_000180655.1_ASM18065v1 | 4,34798 | 2009-06-22 | 0 | 0 | 0 | 36 | 0 |
| Amborella trichopoda | GCA_000471905.1_AMTR1.0 | 706,495 | 2013-09-27 | 0 | 0 | 0 | 0 | 0 |
| Ananas comosus | GCA_001540865.1_ASM154086v1 | 382,056 | 2016-01-25 | 1 | 0 | 0 | 0 | 0 |
| Ananas comosus | GCA_001661175.1_ACMD2v1.0 | 524,07 | 2016-06-10 | 0 | 0 | 0 | 0 | 0 |
| Apostasia shenzhenica | GCA_002786265.1_ASM278626v1 | 348,733 | 2017-11-17 | 3 | 0 | 0 | 0 | 0 |
| Aquilaria agallochum | GCA_000696445.1_Aquilaria_agallocha_v1 | 726,71 | 2014-05-29 | 0 | 0 | 0 | 0 | 0 |
| Aquilegia coerulea | GCA_002738505.1_Aquilegia_coerulea_v1 | 301,98 | 2017-10-31 | 0 | 0 | 0 | 0 | 0 |
| Arabidopsis halleri | GCA_003711535.1_GDC_Ahal_v1.0 | 164,574 | 2018-11-05 | 1 | 0 | 0 | 4 | 0 |
| Arabidopsis halleri subsp. gemmifera | GCA_000523005.1_Ahal_1.0 | 221,14 | 2014-01-16 | 147 | 0 | 9 | 941 | 0 |
| Arabidopsis halleri subsp. gemmifera | GCA_900078215.1_Ahal2.2 | 196,243 | 2016-09-29 | 0 | 0 | 0 | 1 | 0 |
| Arabidopsis halleri subsp. gemmifera | GCA_003118655.1_Ahalleri_gemmifera_v1 | 413,881 | 2018-03-21 | 73 | 0 | 1 | 282 | 0 |
| Arabidopsis lyrata subsp. lyrata | GCA_000004255.1_v.1.0 | 206,823 | 2009-11-30 | 0 | 0 | 0 | 0 | 0 |
| Arabidopsis lyrata subsp. petraea | GCA_000524985.1_Alyr_1.0 | 202,972 | 2014-01-16 | 116 | 0 | 6 | 856 | 0 |
| Arabidopsis lyrata subsp. petraea | GCA_900205625.1_Alyrpet2.2 | 175,183 | 2018-08-06 | 0 | 0 | 1 | 0 | 0 |
| Arabidopsis thaliana | GCA_000211275.1_ASM21127v1 | 93,6545 | 2000-12-14 | 0 | 0 | 0 | 0 | 0 |
| Arabidopsis thaliana | GCA_000001735.2_TAIR10.1 | 119,669 | 2001-08-13 | 0 | 0 | 0 | 0 | 0 |
| Arabidopsis thaliana | GCA_000222325.1_Bur-0_2010-09-30 | 96,5002 | 2011-07-18 | 0 | 0 | 0 | 0 | 0 |
| Arabidopsis thaliana | GCA_000222345.1_C24_2010-09-30 | 98,0662 | 2011-07-18 | 0 | 0 | 0 | 0 | 0 |
| Arabidopsis thaliana | GCA_000222365.1_Ler-1_2010-09-30 | 96,2565 | 2011-07-18 | 0 | 0 | 0 | 0 | 0 |
| Arabidopsis thaliana | GCA_000222385.1_Kro-0_2010-09-30 | 96,694 | 2011-07-18 | 0 | 0 | 0 | 0 | 0 |
| Arabidopsis thaliana | GCA_000835945.1_ASM83594v1 | 127,419 | 2014-11-10 | 0 | 0 | 0 | 0 | 0 |
| Arabidopsis thaliana | GCA_001651475.1_Ler_Assembly | 118,891 | 2016-05-25 | 0 | 0 | 0 | 2 | 0 |
| Arabidopsis thaliana | GCA_001742845.1_AthNd1_v1.0 | 116,846 | 2016-09-23 | 2 | 0 | 0 | 5 | 0 |
| Arabidopsis thaliana | GCA_001753755.2_Athal_Col0Cvi0F1_phased_diploid_1.0 | 244,583 | 2016-10-07 | 1 | 0 | 0 | 122 | 0 |
| Arabidopsis thaliana | GCA_900303345.1_PBfal_IT4 | 119,75 | 2018-03-09 | 0 | 0 | 0 | 0 | 0 |
| Arabidopsis thaliana | GCA_900303355.1_ONTmin_IT4 | 119,503 | 2018-03-09 | 0 | 0 | 0 | 0 | 0 |
| Arabidopsis thaliana | GCA_900243935.1_Col0_oxford_assembly | 119,203 | 2018-03-18 | 0 | 0 | 0 | 0 | 0 |
| Arabidopsis thaliana | GCA_900243945.1_SAIL_oxford_assembly | 119,167 | 2018-03-19 | 0 | 0 | 0 | 6 | 0 |
| Arabidopsis thaliana | GCA_900243955.1_SALK_oxford_assembly | 119,128 | 2018-03-19 | 0 | 0 | 0 | 3 | 0 |
| Arabis alpina | GCA_000612745.1_AA1 | 171,788 | 2014-04-01 | 0 | 0 | 0 | 0 | 0 |
| Arabis alpina | GCA_000733195.1_A_alpina_V4 | 308,033 | 2014-07-21 | 2 | 0 | 0 | 2 | 0 |
| Arabis alpina | GCA_900128785.1_MPIPZ.v5 | 311,642 | 2016-12-31 | 0 | 0 | 0 | 0 | 0 |
| Arabis montbretiana | GCA_001484125.1_ASM148412v1 | 199,12 | 2016-01-04 | 0 | 0 | 0 | 29 | 0 |
| Arabis nordmanniana | GCA_001484925.1_ASM148492v1 | 342,307 | 2016-01-04 | 2 | 0 | 1 | 1532 | 0 |
| Arachis duranensis | GCA_000817695.2_Aradu1.1 | 1084,26 | 2015-01-13 | 0 | 0 | 0 | 0 | 0 |
| Arachis duranensis | GCA_001687015.1_ASM168701v1 | 1075,96 | 2016-07-18 | 39 | 0 | 2 | 482 | 1 |
| Arachis hypogaea | GCA_003086295.1_arahy.Tifrunner.gnm1.KYV3 | 2538,44 | 2018-05-02 | 0 | 0 | 0 | 1 | 0 |
| Arachis hypogaea | GCA_003713155.1_ASM371315v1 | 2506,71 | 2018-07-20 | 0 | 0 | 0 | 0 | 0 |
| Arachis ipaensis | GCA_000816755.2_Araip1.1 | 1353,5 | 2014-12-11 | 0 | 0 | 0 | 0 | 0 |
| Arachis monticola | GCA_003063285.2_ASM306328v2 | 2618,65 | 2018-04-23 | 1 | 0 | 0 | 13 | 0 |
| Argania spinosa | GCA_003260245.1_arg_spin_01 | 670,097 | 2018-06-22 | 8 | 0 | 0 | 46 | 0 |
| Artemisia annua | GCA_003112345.1_ASM311234v1 | 1792,86 | 2018-05-08 | 0 | 0 | 0 | 0 | 0 |
| Artocarpus camansi | GCA_002024485.1_Acamansi1.0 | 631,308 | 2017-03-09 | 3 | 0 | 0 | 375 | 0 |
| Asclepias syriaca | GCA_002018285.1_ASM201828v1 | 236,77 | 2017-03-03 | 3 | 0 | 0 | 41 | 0 |
| Asparagus officinalis | GCA_001876935.1_Aspof.V1 | 1187,54 | 2016-11-09 | 6 | 0 | 0 | 0 | 2 |
| Atalantia buxifolia | GCA_002013935.1_ASM201393v1 | 315,806 | 2017-03-03 | 1 | 0 | 0 | 0 | 0 |
| Auxenochlorella protothecoides | GCA_000733215.1_ASM73321v1 | 22,9246 | 2014-07-23 | 0 | 0 | 0 | 0 | 0 |
| Auxenochlorella protothecoides | GCA_002154875.1_AuxProt1.0 | 32,73 | 2017-05-22 | 0 | 0 | 0 | 0 | 0 |
| Auxenochlorella protothecoides | GCA_003709365.1_ASM370936v1 | 21,2176 | 2018-11-01 | 0 | 0 | 0 | 0 | 0 |
| Auxenochlorella pyrenoidosa | GCA_001430745.1_ASM143074v1 | 56,993 | 2015-11-03 | 0 | 0 | 0 | 0 | 0 |
| Avena sativa | GCA_002943605.1_RSPBVictoria1.0 | 67,3266 | 2018-02-26 | 1 | 0 | 0 | 1 | 0 |
| Azadirachta indica | GCA_000439995.3_AzaInd2.1 | 261,458 | 2013-07-25 | 2 | 0 | 0 | 8 | 0 |
| Barbarea vulgaris | GCA_001920985.1_ASM192098v1 | 167,352 | 2016-12-23 | 0 | 0 | 0 | 0 | 0 |
| Bathycoccus prasinos | GCA_002220235.1_ASM222023v1 | 15,0743 | 2012-06-21 | 0 | 0 | 0 | 0 | 0 |
| Bathycoccus sp. TOSAG39-1 | GCA_900128745.1_TOSAG39-1 | 10,0579 | 2016-11-26 | 0 | 0 | 0 | 0 | 0 |
| Begonia fuchsioides | GCA_003255005.1_ASM325500v1 | 373,914 | 2018-06-19 | 8 | 0 | 0 | 6 | 0 |
| Berberis thunbergii | GCA_003290165.1_B.thun_GenomeAssembly_v1 | 2240,74 | 2018-07-06 | 1 | 0 | 0 | 0 | 0 |
| Beta vulgaris subsp. vulgaris | GCA_000397105.1_BvvSeq-1 | 426,675 | 2013-05-13 | 441 | 0 | 1 | 6 | 2 |
| Beta vulgaris subsp. vulgaris | GCA_000510365.1_KDHBv-1.0.1 | 484,231 | 2013-12-17 | 3 | 0 | 0 | 0 | 0 |
| Beta vulgaris subsp. vulgaris | GCA_000510465.1_UMSBv-1.0.1 | 539,552 | 2013-12-17 | 7 | 0 | 0 | 1 | 0 |
| Beta vulgaris subsp. vulgaris | GCA_000510485.1_YMoBv-1.0.1 | 463,706 | 2013-12-17 | 5 | 0 | 0 | 2 | 0 |
| Beta vulgaris subsp. vulgaris | GCA_000510975.1_RefBeet-1.1.1 | 568,609 | 2013-12-17 | 13 | 0 | 0 | 139 | 0 |
| Beta vulgaris subsp. vulgaris | GCA_000510875.1_YTiBv-1.0.1 | 479,876 | 2013-12-18 | 6 | 0 | 0 | 210 | 0 |
| Beta vulgaris subsp. vulgaris | GCA_000511025.2_RefBeet-1.2.2 | 566,55 | 2013-12-18 | 13 | 0 | 0 | 132 | 0 |
| Beta vulgaris subsp. vulgaris | GCA_000729925.1_BvulgarisC0t1.0 | 1,15347 | 2014-07-16 | 0 | 0 | 7 | 232 | 0 |
| Beta vulgaris subsp. vulgaris | GCA_002917755.1_EL10_1.0 | 540,534 | 2018-02-05 | 7 | 0 | 0 | 0 | 0 |
| Betula nana | GCA_000327005.1_ASM32700v1 | 564,011 | 2012-11-28 | 39 | 0 | 0 | 67 | 0 |
| Betula pendula | GCA_900184695.1_Bpev01 | 435,915 | 2017-05-25 | 0 | 0 | 0 | 243 | 0 |
| Boechera stricta | GCA_002079875.1_Bstricta_278_v1 | 188,795 | 2017-04-05 | 0 | 0 | 0 | 0 | 0 |
| Boehmeria nivea | GCA_002806895.1_ASM280689v1 | 316,026 | 2017-12-04 | 5 | 0 | 0 | 119 | 0 |
| Boehmeria nivea | GCA_002937015.1_ASM293701v1 | 344,617 | 2018-02-21 | 0 | 0 | 0 | 0 | 0 |
| Botryococcus braunii | GCA_002005505.1_B_braunii_Showa_v1 | 184,382 | 2017-02-22 | 1 | 0 | 0 | 1 | 0 |
| Brachypodium distachyon | GCA_000005505.4_Brachypodium_distachyon_v3.0 | 271,299 | 2010-02-10 | 0 | 0 | 0 | 0 | 0 |
| Brachypodium distachyon | GCA_001742125.1_ASM174212v1 | 214,716 | 2016-09-22 | 144 | 0 | 0 | 0 | 0 |
| Brachypodium distachyon | GCA_002892295.1_ASM289229v1 | 218,015 | 2018-01-18 | 186 | 0 | 0 | 2 | 0 |
| Brachypodium distachyon | GCA_002892335.1_ASM289233v1 | 218,676 | 2018-01-18 | 183 | 0 | 0 | 2 | 0 |
| Brassica cretica | GCA_003260635.1_B_cretica_B_v1 | 208,354 | 2018-06-22 | 0 | 0 | 0 | 0 | 0 |
| Brassica cretica | GCA_003260655.1_B_cretica_A_v1 | 412,521 | 2018-06-22 | 0 | 0 | 0 | 6 | 0 |
| Brassica cretica | GCA_003260675.1_B_cretica_C_v1 | 434,935 | 2018-06-22 | 0 | 0 | 0 | 0 | 0 |
| Brassica cretica | GCA_003260695.1_B_cretica_D_v1 | 400,212 | 2018-06-22 | 2 | 0 | 0 | 2 | 0 |
| Brassica juncea var. tumida | GCA_001687265.1_Brassica_juncea_var_tumida_T84-66_v1 | 954,861 | 2016-07-19 | 0 | 0 | 0 | 0 | 0 |
| Brassica napus | GCA_000686985.2_Bra_napus_v2.0 | 976,191 | 2014-05-05 | 6 | 0 | 0 | 7 | 0 |
| Brassica napus | GCA_000751015.1_AST_PRJEB5043_v1 | 848,2 | 2014-08-22 | 30 | 0 | 0 | 5 | 0 |
| Brassica nigra | GCA_001682895.1_ASM168289v1 | 402,145 | 2016-07-10 | 4 | 0 | 0 | 148 | 0 |
| Brassica oleracea | GCA_900416815.2_Brassica_oleracea_HDEM | 554,977 | 2018-05-26 | 0 | 0 | 0 | 0 | 0 |
| Brassica oleracea var. capitata | GCA_000604025.1_BOL_v1.0 | 514,431 | 2014-03-28 | 0 | 0 | 0 | 27 | 0 |
| Brassica oleracea var. oleracea | GCA_000695525.1_BOL | 488,954 | 2014-05-22 | 2 | 0 | 0 | 24 | 0 |
| Brassica rapa | GCA_000309985.1_Brapa_1.0 | 284,129 | 2011-07-14 | 7 | 0 | 0 | 5 | 0 |
| Brassica rapa | GCA_900412535.2_Brassica_rapa_Z1 | 401,927 | 2018-05-26 | 0 | 0 | 0 | 0 | 0 |
| Brassica rapa | GCA_003434825.1_Brassica_rapa_FPsc_v1.3 | 314,865 | 2018-08-30 | 0 | 0 | 0 | 0 | 0 |
| Cajanus cajan | GCA_000230855.2_Pigeonpea-Version-2.0 | 648,281 | 2011-10-17 | 155 | 0 | 4 | 18332 | 0 |
| Cajanus cajan | GCA_000340665.1_C.cajan_V1.0 | 592,971 | 2013-02-13 | 2 | 0 | 0 | 4648 | 0 |
| Calamus simplicifolius | GCA_900491605.1_Calamus_simplicifolius | 1960,81 | 2018-06-22 | 1 | 0 | 0 | 4 | 0 |
| Camelina sativa | GCA_000496875.1_CamelinaSativa | 547,649 | 2013-11-14 | 0 | 0 | 0 | 0 | 0 |
| Camelina sativa | GCA_000633955.1_Cs | 641,356 | 2014-04-17 | 0 | 0 | 0 | 0 | 0 |
| Cannabis sativa | GCA_000230575.3_ASM23057v3 | 891,965 | 2011-10-13 | 0 | 0 | 0 | 0 | 0 |
| Cannabis sativa | GCA_001509995.1_Chemdog91_175268 | 285,933 | 2016-01-11 | 0 | 0 | 0 | 0 | 0 |
| Cannabis sativa | GCA_001865755.1_ASM186575v1 | 585,824 | 2016-11-03 | 0 | 0 | 0 | 1 | 0 |
| Cannabis sativa | GCA_002090435.1_ASM209043v1 | 512,174 | 2017-04-13 | 1 | 0 | 0 | 2 | 0 |
| Cannabis sativa | GCA_003417725.2_ASM341772v2 | 1009,67 | 2018-08-22 | 0 | 0 | 0 | 0 | 0 |
| Cannabis sativa | GCA_003660325.1_MGC_Can.sativa_JamaicanLion_DASH | 1032,59 | 2018-10-15 | 0 | 0 | 0 | 0 | 0 |
| Cannabis sativa subsp. indica | GCA_001510005.1_ASM151000v1 | 595,358 | 2016-01-11 | 1 | 0 | 0 | 1 | 0 |
| Capsella bursa-pastoris | GCA_001974645.1_C_bursa_pastoris_nuclear_genome_v1 | 268,431 | 2017-01-29 | 0 | 0 | 0 | 2 | 0 |
| Capsella rubella | GCA_000375325.1_Caprub1_0 | 133,064 | 2013-04-19 | 0 | 0 | 0 | 0 | 0 |
| Capsicum annuum | GCA_000512255.2_ASM51225v2 | 3063,86 | 2013-12-30 | 2 | 0 | 0 | 9 | 1 |
| Capsicum annuum | GCA_000710875.1_Pepper_Zunla_1_Ref_v1.0 | 2935,88 | 2014-06-23 | 9 | 0 | 0 | 3 | 0 |
| Capsicum annuum | GCA_002878395.2_UCD10Xv1.0 | 3212,12 | 2017-12-19 | 0 | 0 | 0 | 1 | 0 |
| Capsicum annuum var. glabriusculum | GCA_000950795.1_Pepper_Chiltepin_Ref_v1.0 | 2768,13 | 2014-06-25 | 5 | 0 | 0 | 0 | 0 |
| Capsicum baccatum | GCA_002271885.2_ASM227188v2 | 3215,61 | 2017-08-16 | 1 | 0 | 0 | 1 | 0 |
| Capsicum chinense | GCA_002271895.2_ASM227189v2 | 3070,91 | 2017-08-14 | 0 | 0 | 0 | 44 | 0 |
| Carica papaya | GCA_000150535.1_Papaya1.0 | 370,419 | 2008-04-23 | 2 | 0 | 3 | 2 | 0 |
| Carica papaya | GCA_001310045.1_ASM131004v1 | 8,07745 | 2015-10-09 | 0 | 0 | 0 | 0 | 0 |
| Carnegiea gigantea | GCA_002740515.1_SGP5_Cgig_v1.3 | 980,351 | 2017-10-31 | 0 | 0 | 0 | 0 | 0 |
| Carthamus tinctorius | GCA_001633085.1_Safflower1 | 661,938 | 2016-04-27 | 0 | 0 | 0 | 0 | 0 |
| Castanea mollissima | GCA_000763605.1_ASM76360v1 | 833,241 | 2014-10-03 | 14 | 0 | 0 | 679 | 0 |
| Casuarina glauca | GCA_003255045.1_ASM325504v1 | 282,811 | 2018-06-19 | 0 | 0 | 0 | 0 | 0 |
| Catharanthus roseus | GCA_000949345.1_ASM94934v1 | 522,654 | 2015-03-06 | 0 | 0 | 0 | 0 | 0 |
| Catharanthus roseus | GCA_001292525.1_BAC_T16H | 0,11552 | 2015-09-11 | 0 | 0 | 0 | 0 | 0 |
| Catharanthus roseus | GCA_001292565.1_BAC_SGD | 0,114931 | 2015-09-11 | 0 | 0 | 0 | 0 | 0 |
| Cenchrus americanus | GCA_002174835.2_ASM217483v2 | 1816,95 | 2017-06-06 | 11 | 0 | 0 | 787 | 0 |
| Cephalotus follicularis | GCA_001972305.1_Cfol_1.0 | 1614,52 | 2017-01-11T21:19:00Z | 1 | 0 | 0 | 0 | 0 |
| Cercis canadensis | GCA_003255065.1_ASM325506v1 | 329,325 | 2018-06-19 | 0 | 0 | 0 | 0 | 0 |
| Chamaecrista fasciculata | GCA_003254925.1_ASM325492v1 | 429,103 | 2018-06-19 | 3 | 0 | 0 | 22 | 0 |
| Chara braunii | GCA_003427395.1_Cbr_1.0 | 1751,21 | 2018-07-13T21:44:00Z | 0 | 0 | 0 | 6 | 0 |
| Chenopodium pallidicaule | GCA_001687005.1_ASM168700v1 | 337,011 | 2016-07-18 | 7 | 0 | 0 | 1 | 0 |
| Chenopodium quinoa | GCA_001683475.1_ASM168347v1 | 1333,55 | 2016-07-11 | 0 | 0 | 0 | 0 | 0 |
| Chenopodium quinoa | GCA_001742885.1_Cqu_r1.0 | 1087,41 | 2016-07-27T21:22:00Z | 0 | 0 | 0 | 0 | 0 |
| Chenopodium quinoa | GCA_002732095.1_Cq_real_v1.0 | 1336,74 | 2017-10-18 | 4 | 0 | 0 | 88 | 0 |
| Chenopodium suecicum | GCA_001687025.1_ASM168702v1 | 536,949 | 2016-07-18 | 0 | 0 | 0 | 0 | 0 |
| Chlamydomonas applanata | GCA_001662365.1_Cap_assembly01 | 78,5042 | 2016-04-22 | 0 | 0 | 0 | 0 | 0 |
| Chlamydomonas asymmetrica | GCA_001662385.1_Cas_assembly01 | 141,916 | 2016-04-22 | 0 | 0 | 0 | 0 | 0 |
| Chlamydomonas debaryana | GCA_001662405.1_Cde_assembly01 | 120,364 | 2016-04-22 | 0 | 0 | 0 | 2 | 0 |
| Chlamydomonas eustigma | GCA_002335675.1_C.eustigma_genome_v1.0 | 66,6295 | 2017-08-31T20:51:00Z | 0 | 0 | 0 | 0 | 0 |
| Chlamydomonas reinhardtii | GCA_000002595.2_v3.0 | 120,405 | 2007-08-03 | 0 | 0 | 0 | 143 | 0 |
| Chlamydomonas sphaeroides | GCA_001662425.1_Csp_assembly01 | 122,189 | 2016-04-22 | 0 | 0 | 0 | 0 | 0 |
| Chlorella sorokiniana | GCA_002245835.2_Chlorella_sorokiniana_2.0 | 59,5662 | 2017-08-10 | 0 | 0 | 0 | 0 | 0 |
| Chlorella sorokiniana | GCA_002939045.1_CSI_1228 | 61,3911 | 2018-02-23 | 0 | 0 | 0 | 0 | 0 |
| Chlorella sorokiniana | GCA_003116155.1_ASM311615v1 | 57,8816 | 2018-05-11 | 0 | 0 | 0 | 0 | 0 |
| Chlorella sorokiniana | GCA_003130725.1_ASM313072v1 | 58,5349 | 2018-05-17 | 0 | 0 | 0 | 0 | 0 |
| Chlorella sp. A99 | GCA_003063905.1_ASM306390v1 | 40,934 | 2018-04-23 | 0 | 0 | 0 | 0 | 0 |
| Chlorella sp. ArM0029B | GCA_002896455.3_ArM29Bkp_1312 | 92,9613 | 2018-01-24 | 0 | 0 | 0 | 0 | 0 |
| Chlorella variabilis | GCA_000147415.1_v_1.0 | 46,1595 | 2010-08-31 | 0 | 0 | 0 | 0 | 0 |
| Chlorella vulgaris | GCA_001021125.1_ASM102112v1 | 37,3422 | 2015-06-05 | 0 | 0 | 0 | 0 | 0 |
| Cicer arietinum | GCA_000331145.1_ASM33114v1 | 530,894 | 2012-12-31 | 7 | 0 | 0 | 5 | 0 |
| Cicer arietinum | GCA_000347275.3_ASM34727v3 | 510,877 | 2013-03-15 | 111 | 0 | 1 | 631 | 2 |
| Cicer arietinum | GCA_002896005.1_CDCFrontier_v0.3 | 644,322 | 2018-01-23 | 0 | 0 | 0 | 0 | 0 |
| Cicer echinospermum | GCA_002896215.1_S2Drd065_v0.3 | 644,721 | 2018-01-23 | 0 | 0 | 0 | 0 | 0 |
| Cicer reticulatum | GCA_002896235.1_Besev079_v0.3 | 715,407 | 2018-01-23 | 0 | 0 | 0 | 0 | 0 |
| Cicer reticulatum | GCA_003689015.1_ASM368901v1 | 416,688 | 2018-10-25 | 1 | 0 | 0 | 37 | 0 |
| Cinnamomum micranthum f. kanehirae | GCA_003546025.1_ASBRC_Ckan_1.0 | 730,416 | 2018-09-11 | 4 | 0 | 0 | 0 | 0 |
| Cissus quadrangularis | GCA_002878655.1_ASM287865v1 | 281,704 | 2018-01-12 | 0 | 0 | 0 | 0 | 0 |
| Citrullus lanatus | GCA_000238415.1_CiLa_1.0 | 321,047 | 2011-12-13 | 0 | 0 | 0 | 0 | 0 |
| Citrus cavaleriei | GCA_002013975.2_ASM201397v2 | 357,621 | 2017-03-03 | 2 | 0 | 0 | 17 | 0 |
| Citrus clementina | GCA_000493195.1_Citrus_clementina_v1.0 | 301,365 | 2013-10-30 | 0 | 0 | 0 | 0 | 0 |
| Citrus maxima | GCA_002006925.1_ASM200692v1 | 345,757 | 2017-02-24 | 2 | 0 | 0 | 6 | 0 |
| Citrus medica | GCA_002013955.2_C_medica_denovo_2 | 406,058 | 2017-03-03 | 2 | 0 | 0 | 5 | 0 |
| Citrus reticulata | GCA_003258625.1_ASM325862v1 | 344,273 | 2018-06-20 | 0 | 0 | 0 | 0 | 0 |
| Citrus sinensis | GCA_000317415.1_Csi_valencia_1.0 | 327,83 | 2012-09-18 | 24 | 0 | 0 | 3 | 1 |
| Citrus sinensis | GCA_000695605.1_Citrus_sinensis_v1.0 | 319,225 | 2014-05-22 | 0 | 0 | 0 | 0 | 0 |
| Citrus unshiu | GCA_001753815.1_CunshiuBMS10_01 | 1,1542 | 2016-10-03T20:44:00Z | 0 | 0 | 0 | 0 | 0 |
| Citrus unshiu | GCA_002897195.1_CUMW_v1.0 | 359,652 | 2017-12-06 | 1 | 0 | 0 | 0 | 0 |
| Citrus x paradisi x Citrus trifoliata | GCA_001929425.1_WD23_11_assembly_v1 | 265,534 | 2017-01-03 | 2 | 0 | 6 | 5 | 0 |
| Coccomyxa sp. LA000219 | GCA_000812005.1_ASM81200v1 | 48,5465 | 2014-12-30 | 0 | 0 | 0 | 0 | 0 |
| Coccomyxa sp. SUA001 | GCA_001244535.1_ASM124453v1 | 11,7544 | 2015-08-05 | 0 | 0 | 0 | 0 | 0 |
| Coccomyxa subellipsoidea C-169 | GCA_000258705.1_Coccomyxa_subellipsoidae_v2.0 | 48,8266 | 2012-04-13 | 0 | 0 | 0 | 0 | 0 |
| Cocos nucifera | GCA_003604295.1_ASM360429v1 | 1839,17 | 2018-10-01 | 0 | 0 | 0 | 0 | 0 |
| Coelastrella sp. M60 | GCA_001630525.1_ASM163052v1 | 80,2218 | 2016-04-25 | 0 | 0 | 0 | 41 | 0 |
| Coelastrella sp. UTEX B 3026 | GCA_002588565.1_ASM258856v1 | 151,547 | 2017-10-18 | 0 | 0 | 0 | 0 | 0 |
| Coffea arabica | GCA_003713225.1_Cara_1.0 | 1094,29 | 2018-11-06 | 3 | 0 | 0 | 0 | 0 |
| Coffea eugenioides | GCA_003713205.1_Ceug_1.0 | 699,904 | 2018-11-06 | 0 | 0 | 0 | 0 | 0 |
| Conringia planisiliqua | GCA_900108845.1_Conringia_planisiliqua.v1 | 184,156 | 2016-12-31 | 0 | 0 | 0 | 0 | 0 |
| Corchorus capsularis | GCA_001974805.1_CCACVL1_1.0 | 317,178 | 2017-01-30 | 0 | 0 | 0 | 57 | 0 |
| Corchorus olitorius | GCA_001974825.1_COLO4_1.0 | 334,912 | 2017-01-30 | 2 | 0 | 0 | 815 | 0 |
| Corchorus olitorius | GCA_002141455.1_Co_S7_2014 | 377,377 | 2017-05-16 | 0 | 0 | 0 | 1030 | 0 |
| Cucumis melo | GCA_000313045.1_ASM31304v1 | 374,928 | 2012-06-29 | 92 | 0 | 0 | 13 | 1 |
| Cucumis sativus | GCA_000004075.2_ASM407v2 | 195,669 | 2009-10-20 | 0 | 0 | 0 | 5 | 0 |
| Cucumis sativus | GCA_000224045.1_CSB10A_v1 | 323,986 | 2011-08-18 | 0 | 0 | 0 | 0 | 0 |
| Cucumis sativus | GCA_001483825.2_B10v2 | 342,654 | 2015-12-31 | 10 | 0 | 0 | 0 | 0 |
| Cucurbita maxima | GCA_002738345.1_Cmax_1.0 | 271,413 | 2017-10-31 | 0 | 0 | 0 | 0 | 0 |
| Cucurbita moschata | GCA_002738365.1_Cmos_1.0 | 269,943 | 2017-10-31 | 26 | 0 | 0 | 2 | 0 |
| Cucurbita pepo subsp. pepo | GCA_002806865.2_ASM280686v2 | 261,355 | 2017-12-04 | 1 | 0 | 0 | 169 | 0 |
| Cuscuta australis | GCA_003260385.1_Cau_v1.0 | 262,63 | 2018-06-22 | 7 | 0 | 0 | 0 | 0 |
| Cuscuta campestris | GCA_900332095.1_ASM90033209v1 | 476,792 | 2018-04-12 | 0 | 0 | 0 | 0 | 0 |
| Cymbomonas tetramitiformis | GCA_001247695.1_ASM124769v1 | 281,27 | 2015-08-05 | 0 | 0 | 0 | 0 | 0 |
| Cynara cardunculus var. scolymus | GCA_001531365.1_CcrdV1 | 725,198 | 2016-01-21 | 0 | 0 | 0 | 0 | 0 |
| Dactylis glomerata | GCA_002892645.1_ASM289264v1 | 839,915 | 2018-01-19 | 33 | 0 | 0 | 18 | 0 |
| Datisca glomerata | GCA_003255025.1_ASM325502v1 | 688,404 | 2018-06-19 | 24 | 0 | 0 | 1 | 0 |
| Daucus carota subsp. sativus | GCA_001625215.1_ASM162521v1 | 421,539 | 2016-04-20 | 0 | 0 | 0 | 2 | 0 |
| Dendrobium officinale | GCA_001605985.1_ASM160598v1 | 1008,68 | 2016-02-26 | 2 | 0 | 0 | 0 | 0 |
| Dianthus caryophyllus | GCA_000512335.1_DCA_r1.0 | 567,662 | 2013-12-18 | 0 | 0 | 0 | 5 | 0 |
| Dichanthelium oligosanthes | GCA_001633215.2_ASM163321v2 | 589,166 | 2016-04-28 | 52 | 0 | 0 | 1 | 0 |
| Dioscorea alata | GCA_002904275.2_ASM290427v2 | 620,909 | 2015-10-10 | 0 | 0 | 0 | 0 | 0 |
| Dioscorea rotundata | GCA_002240015.2_TDr96_F1_Pseudo_Chromosome_v1.0 | 456,675 | 2017-07-28 | 0 | 0 | 0 | 0 | 0 |
| Dioscorea rotundata | GCA_002260605.1_TDr96x99_v1.0.fasta | 594,227 | 2017-07-28 | 1 | 0 | 0 | 1 | 0 |
| Dioscorea rotundata | GCA_002260645.1_TDr97_00777_Male_DDN | 683,283 | 2017-07-28 | 317 | 0 | 3 | 126 | 3 |
| Dioscorea rotundata | GCA_002260665.1_TDr97_00917_Female_DDN | 730,21 | 2017-07-28 | 160 | 0 | 0 | 254 | 2 |
| Diospyros lotus | GCA_000774125.1_ASM77412v1 | 1,10419 | 2014-11-10 | 0 | 0 | 0 | 0 | 0 |
| Dorcoceras hygrometricum | GCA_001598015.1_Boea_hygrometrica.v1 | 1521,36 | 2016-03-25 | 5 | 0 | 0 | 62 | 0 |
| Drosera capensis | GCA_001925005.1_ASM192500v1 | 263,788 | 2016-12-30 | 0 | 0 | 0 | 6 | 0 |
| Dryas drummondii | GCA_003254865.1_ASM325486v1 | 225,547 | 2018-06-19 | 12 | 0 | 0 | 65 | 0 |
| Dunaliella salina | GCA_002284615.1_Dsal_v1.0 | 343,704 | 2017-08-31 | 0 | 0 | 0 | 0 | 0 |
| Durio zibethinus | GCA_002303985.1_Duzib1.0 | 715,23 | 2017-09-15 | 1 | 0 | 0 | 0 | 0 |
| Echinochloa crus-galli | GCA_900205405.1_ASM90020540v1 | 1486,61 | 2017-10-23 | 114 | 0 | 1 | 15 | 3 |
| Echium plantagineum | GCA_003412495.1_ASM341249v1 | 348,921 | 2018-08-21 | 0 | 0 | 0 | 0 | 0 |
| Eichhornia paniculata | GCA_001647135.1_ASM164713v1 | 571,388 | 2016-05-02 | 0 | 0 | 0 | 0 | 0 |
| Elaeis guineensis | GCA_000442705.1_EG5 | 1535,18 | 2013-07-26 | 0 | 0 | 0 | 19 | 0 |
| Elaeis guineensis | GCA_001672495.1_ASM167249v1 | 499,029 | 2016-06-20 | 6 | 0 | 0 | 0 | 0 |
| Elaeis guineensis | GCA_002146295.1_EG01 | 134,97 | 2017-05-17 | 48 | 0 | 0 | 13 | 0 |
| Elaeis oleifera | GCA_000441515.1_EO8 | 1402,73 | 2013-07-25 | 0 | 0 | 0 | 0 | 0 |
| Elaeis oleifera | GCA_002146275.1_EO01 | 60,0019 | 2017-05-17 | 10 | 0 | 0 | 5 | 0 |
| Eleusine coracana subsp. coracana | GCA_002180455.1_ASM218045v1 | 1195,99 | 2017-06-08 | 3056 | 0 | 0 | 25790 | 0 |
| Embelia ribes | GCA_001753735.1_Embelia_ribes_ER1_v1 | 660,51 | 2016-10-06 | 0 | 0 | 0 | 0 | 0 |
| Ensete ventricosum | GCA_000331365.2_Ensete_JungleSeeds_v2.0 | 437,27 | 2013-01-07 | 0 | 0 | 0 | 0 | 0 |
| Ensete ventricosum | GCA_000818735.2_Ensete_Bedadeti_v2.0 | 451,284 | 2015-01-09 | 0 | 0 | 0 | 2 | 0 |
| Ensete ventricosum | GCA_001884805.1_Derea_v1.0 | 429,48 | 2016-11-21 | 0 | 0 | 0 | 59 | 0 |
| Ensete ventricosum | GCA_001884845.1_Onjamo_v1.0 | 444,842 | 2016-11-21 | 0 | 0 | 0 | 0 | 0 |
| Eragrostis tef | GCA_000970635.1_ASM97063v1 | 607,318 | 2015-04-08 | 1 | 0 | 0 | 250 | 0 |
| Erigeron canadensis | GCA_000775935.1_ASM77593v1 | 326,165 | 2014-11-13 | 0 | 0 | 0 | 0 | 0 |
| Erythranthe guttata | GCA_000504015.1_Mimgu1_0 | 322,167 | 2013-12-04 | 0 | 0 | 0 | 0 | 0 |
| Eschscholzia californica subsp. californica | GCA_002897215.1_ECA_r1.0 | 489,065 | 2017-12-28 | 0 | 0 | 0 | 0 | 0 |
| Eucalyptus camaldulensis | GCA_000260855.1_EUC_r1.0 | 654,922 | 2012-01-21 | 5 | 0 | 0 | 54 | 0 |
| Eucalyptus grandis | GCA_000612305.1_Egrandis1_0 | 691,43 | 2014-04-02 | 0 | 0 | 0 | 4 | 0 |
| Euclidium syriacum | GCA_900116095.1_Euclidium_syriacum.MPIPZ.v1 | 229,211 | 2016-12-31 | 3 | 0 | 0 | 0 | 0 |
| Eudorina sp. 2006-703-Eu-15 | GCA_003117095.1_EudorinaMale_1.0 | 168,621 | 2018-04-06T21:23:00Z | 29 | 0 | 0 | 0 | 0 |
| Eudorina sp. 2006-703-Eu-15 | GCA_003117195.1_EudorinaFemale_1.0 | 184,032 | 2018-04-06T21:43:00Z | 15 | 0 | 0 | 0 | 0 |
| Euphorbia esula | GCA_002918425.1_ASM291842v1 | 639,02 | 2018-02-06 | 28 | 0 | 0 | 2875 | 1 |
| Euphorbia esula | GCA_002919075.1_ASM291907v1 | 1124,89 | 2018-02-06 | 13 | 0 | 0 | 343 | 0 |
| Eutrema heterophyllum | GCA_002933915.1_ASM293391v1 | 348,971 | 2018-02-20 | 0 | 0 | 0 | 4 | 1 |
| Eutrema salsugineum | GCA_000325905.2_TsV2-8 | 231,893 | 2012-06-29 | 0 | 0 | 0 | 0 | 0 |
| Eutrema salsugineum | GCA_000478725.1_Eutsalg1_0 | 243,11 | 2013-10-22 | 0 | 0 | 0 | 0 | 0 |
| Eutrema yunnanense | GCA_002933935.1_ASM293393v1 | 415,364 | 2018-02-20 | 3 | 0 | 0 | 9 | 0 |
| Fagopyrum esculentum | GCA_001661195.1_FES_r1.0 | 1177,69 | 2016-05-11 | 19 | 0 | 0 | 0 | 0 |
| Fagopyrum tataricum | GCA_002319775.1_Ft1.0 | 505,883 | 2017-09-20 | 1 | 0 | 0 | 36 | 0 |
| Fagopyrum tataricum | GCA_002928575.1_Ft54 | 526,768 | 2018-02-14 | 0 | 0 | 0 | 0 | 0 |
| Fagus sylvatica | GCA_003347535.1_UKW_Fsyl_0.5 | 428,2 | 2018-07-31 | 4 | 0 | 0 | 1 | 0 |
| Ficus carica | GCA_002002945.1_F.carica_assembly01 | 247,091 | 2017-01-27T21:17:00Z | 0 | 0 | 2 | 0 | 0 |
| Foeniculum vulgare | GCA_003724115.1_FoenVul1.0 | 1010,97 | 2018-11-13 | 0 | 0 | 0 | 0 | 0 |
| Fragaria iinumae | GCA_000511975.1_FII_r1.1 | 199,628 | 2013-11-27 | 0 | 0 | 0 | 0 | 0 |
| Fragaria nipponica | GCA_000512025.1_FNI_r1.1 | 206,415 | 2013-11-27 | 0 | 0 | 0 | 118 | 0 |
| Fragaria nubicola | GCA_000511995.1_FNU_r1.1 | 203,686 | 2013-11-27 | 0 | 0 | 0 | 0 | 0 |
| Fragaria orientalis | GCA_000517285.1_FOR_r1.1 | 214,184 | 2013-11-27 | 5 | 0 | 0 | 1 | 0 |
| Fragaria vesca subsp. vesca | GCA_000184155.1_FraVesHawaii_1.0 | 214,373 | 2010-12-22 | 1 | 0 | 0 | 0 | 0 |
| Fragaria x ananassa | GCA_000511695.1_FANhybrid_r1.2 | 173,23 | 2013-11-27 | 0 | 0 | 0 | 3 | 0 |
| Fragaria x ananassa | GCA_000511835.1_FAN_r1.1 | 697,762 | 2013-11-27 | 12 | 0 | 0 | 2 | 1 |
| Fraxinus excelsior | GCA_900149125.1_BATG-0.5 | 867,455 | 2017-01-20 | 3 | 0 | 0 | 32 | 0 |
| Gastrodia elata f. glauca | GCA_002966915.1_ASM296691v1 | 1060,98 | 2018-03-02 | 1 | 0 | 0 | 0 | 0 |
| Genlisea aurea | GCA_000441915.1_GenAur_1.0 | 43,3578 | 2013-07-26 | 0 | 0 | 0 | 0 | 0 |
| Geum urbanum | GCA_900236755.1_G_urb_d1 | 1217,04 | 2018-01-15 | 0 | 0 | 2 | 10 | 0 |
| Glycine max | GCA_000004515.4_Glycine_max_v2.1 | 979,046 | 2010-01-05 | 0 | 0 | 0 | 0 | 0 |
| Glycine max | GCA_001269945.2_Glycine_max_Enrei_2.0 | 927,706 | 2015-07-03 | 345 | 0 | 3 | 70 | 2 |
| Glycine max | GCA_002905335.1_glyma.Lee.gnm1 | 990,714 | 2018-01-29 | 0 | 0 | 0 | 0 | 0 |
| Glycine max | GCA_003349995.1_Gmax_ZH13 | 1017,57 | 2018-07-31 | 0 | 0 | 0 | 0 | 0 |
| Glycine soja | GCA_000722935.2_W05v1.0 | 863,568 | 2014-07-07 | 7 | 0 | 0 | 959 | 0 |
| Glycine soja | GCA_002907465.1_glyso.PI483463.gnm1 | 985,26 | 2018-01-29 | 0 | 0 | 0 | 0 | 0 |
| Gonium pectorale | GCA_001584585.1_ASM158458v1 | 148,806 | 2016-03-09 | 0 | 0 | 0 | 0 | 0 |
| Gossypioides kirkii | GCA_002818315.1_Gokir.v1 | 528,715 | 2017-12-06 | 0 | 0 | 0 | 2 | 0 |
| Gossypium arboreum | GCA_000612285.2_Gossypium_arboreum_v1.0 | 1694,6 | 2014-03-31 | 9 | 0 | 0 | 5 | 0 |
| Gossypium arboreum | GCA_000787975.1_arboreum_v1.0 | 1862,24 | 2014-11-25 | 29 | 0 | 1 | 55 | 1 |
| Gossypium barbadense | GCA_001856525.1_GbV1.0 | 2566,74 | 2016-10-28 | 44 | 0 | 0 | 4 | 0 |
| Gossypium barbadense | GCA_002926015.1_ASM292601v1 | 775,252 | 2018-02-13 | 0 | 0 | 0 | 0 | 0 |
| Gossypium barbadense | GCA_002928715.1_ASM292871v1 | 1394,24 | 2018-02-13 | 0 | 0 | 0 | 0 | 0 |
| Gossypium hirsutum | GCA_000987745.1_ASM98774v1 | 2189,14 | 2015-04-29 | 0 | 0 | 0 | 7 | 0 |
| Gossypium hirsutum | GCA_002504345.1_ASM250434v1 | 169,285 | 2017-10-10 | 0 | 0 | 0 | 0 | 0 |
| Gossypium raimondii | GCA_000327365.1_Graimondii2_0 | 761,565 | 2012-12-20 | 0 | 0 | 0 | 0 | 0 |
| Gossypium raimondii | GCA_000331045.1_Gr_v1.0 | 773,768 | 2013-01-04 | 0 | 0 | 0 | 0 | 0 |
| Handroanthus impetiginosus | GCA_002762385.1_Himp0.1 | 503,289 | 2017-11-09 | 0 | 0 | 0 | 2 | 0 |
| Helianthus annuus | GCA_002127325.1_HanXRQr1.0 | 3027,84 | 2017-05-08 | 17 | 0 | 0 | 3 | 0 |
| Helicosporidium sp. ATCC 50920 | GCA_000690575.1_Helico_v1.0 | 12,3738 | 2014-05-13 | 0 | 0 | 0 | 1 | 0 |
| Herrania umbratica | GCA_002168275.2_ASM216827v2 | 234,039 | 2017-06-05 | 0 | 0 | 0 | 0 | 0 |
| Hevea brasiliensis | GCA_000340545.1_Hevbra_1.0 | 1301,4 | 2013-02-06 | 10 | 0 | 0 | 24 | 0 |
| Hevea brasiliensis | GCA_001654055.1_ASM165405v1 | 1373,53 | 2016-06-01 | 0 | 0 | 0 | 0 | 0 |
| Hevea brasiliensis | GCA_001907995.1_ASM190799v1 | 1550,51 | 2016-12-12 | 1 | 0 | 0 | 0 | 0 |
| Hevea brasiliensis | GCA_002003025.1_HbrasiliensisBPM24_01 | 1256,27 | 2017-01-14 | 82 | 0 | 5 | 77 | 2 |
| Hibiscus syriacus | GCA_001696755.1_ASM169675v1 | 1748,25 | 2016-07-29 | 1 | 0 | 0 | 0 | 0 |
| Hordeum bulbosum | GCA_900070015.1_Hordeum_bulbosum_assembly1 | 1294,87 | 2017-02-21 | 0 | 0 | 0 | 51 | 0 |
| Hordeum pubiflorum | GCA_000582825.1_Hordeum_pubiflorum_assembly1 | 1425,27 | 2013-08-21 | 114 | 0 | 1 | 478 | 0 |
| Hordeum vulgare | GCA_000947855.1_563_E_assembled_ctg_200 | 98,056 | 2015-02-27 | 5 | 0 | 0 | 8417 | 1 |
| Hordeum vulgare | GCA_900075435.2_barley_BACs_2 | 9788,86 | 2016-08-25 | 40 | 0 | 0 | 13 | 0 |
| Hordeum vulgare subsp. vulgare | GCA_000227425.1_Hvul_cvHarunaNijo3HBAC01 | 28,016 | 2011-06-22 | 0 | 0 | 0 | 30 | 0 |
| Hordeum vulgare subsp. vulgare | GCA_002900805.1_ASM290080v1 | 2019,37 | 2012-10-19 | 27 | 0 | 0 | 712 | 0 |
| Hordeum vulgare subsp. vulgare | GCA_000326085.1_ASM32608v1 | 1868,64 | 2012-10-29 | 72 | 0 | 1 | 2228 | 0 |
| Hordeum vulgare subsp. vulgare | GCA_000326125.1_ASM32612v1 | 1779,49 | 2012-10-29 | 3 | 0 | 16 | 14367 | 0 |
| Hordeum vulgare subsp. vulgare | GCA_001077415.1_ASM107741v1 | 1645,58 | 2014-01-02 | 8 | 0 | 0 | 810 | 0 |
| Hordeum vulgare subsp. vulgare | GCA_900002345.1_BW457_CLC_assembly | 1825,17 | 2016-02-27 | 48 | 0 | 0 | 632 | 0 |
| Hordeum vulgare subsp. vulgare | GCA_002943585.1_RSPBBaronesse | 57,9644 | 2018-02-26 | 0 | 0 | 0 | 9 | 0 |
| Humulus lupulus var. cordifolius | GCA_000830395.1_hl_KR_version_1.0.fasta | 2049,21 | 2014-12-11 | 14 | 0 | 0 | 1744 | 0 |
| Humulus lupulus var. lupulus | GCA_000831365.1_hl_SW_version_1.0.fasta | 2049,21 | 2014-12-11 | 14 | 0 | 0 | 1744 | 0 |
| Ipomoea batatas | GCA_900092185.1_Ipomoea_batatas_Version1 | 13,8613 | 2017-08-26 | 12 | 0 | 0 | 152 | 0 |
| Ipomoea batatas | GCA_002525835.2_ipoBat4 | 837,013 | 2017-10-11 | 0 | 0 | 0 | 11 | 0 |
| Ipomoea nil | GCA_001879475.1_Asagao_1.1 | 735,231 | 2016-09-01 | 3 | 0 | 0 | 1 | 0 |
| Ipomoea trifida | GCA_000978395.1_ITR_r1.0 | 512,991 | 2015-03-27 | 16 | 0 | 1 | 0 | 0 |
| Ipomoea trifida | GCA_000981105.1_ITRk_r1.0 | 712,155 | 2015-03-27 | 4 | 0 | 6 | 0 | 0 |
| Ipomoea trifida | GCA_003576665.1_ASM357666v1 | 492,376 | 2018-09-19 | 0 | 0 | 0 | 0 | 0 |
| Ipomoea triloba | GCA_003576645.1_ASM357664v1 | 461,827 | 2018-09-19 | 0 | 0 | 0 | 0 | 0 |
| Jatropha curcas | GCA_000208675.2_JAT_r4.5 | 297,661 | 2011-01-07 | 2 | 0 | 0 | 4 | 0 |
| Jatropha curcas | GCA_000696525.1_JatCur_1.0 | 318,527 | 2014-05-23 | 0 | 0 | 0 | 0 | 0 |
| Juglans cathayensis | GCA_003122765.1_ASM312276v1 | 600,151 | 2018-05-14 | 5 | 0 | 0 | 10 | 0 |
| Juglans hindsii | GCA_003123825.1_ASM312382v1 | 611,109 | 2018-05-14 | 36 | 0 | 0 | 124 | 0 |
| Juglans mandshurica | GCA_002916435.1_m4v1 | 558,071 | 2018-02-05 | 3 | 0 | 0 | 1 | 0 |
| Juglans microcarpa | GCA_003123845.1_ASM312384v1 | 913,972 | 2018-05-14 | 62 | 0 | 0 | 191 | 0 |
| Juglans nigra | GCA_002916485.1_n1v1 | 682,557 | 2018-02-05 | 1 | 0 | 0 | 6 | 0 |
| Juglans nigra | GCA_003123865.1_ASM312386v1 | 620,767 | 2018-05-14 | 45 | 0 | 0 | 197 | 0 |
| Juglans regia | GCA_001411555.1_wgs.5d | 699,673 | 2015-10-22 | 441 | 0 | 0 | 1501 | 0 |
| Juglans regia | GCA_002916465.1_r1v1 | 634,748 | 2018-02-05 | 4 | 0 | 0 | 35 | 1 |
| Juglans regia | GCA_003122785.1_ASM312278v1 | 650,478 | 2018-05-14 | 1 | 0 | 0 | 0 | 0 |
| Juglans sigillata | GCA_003123805.1_ASM312380v1 | 648,117 | 2018-05-14 | 3 | 0 | 0 | 55 | 0 |
| Kalanchoe fedtschenkoi | GCA_002312845.1_K_fedtschenkoi_M2_v1 | 256,351 | 2017-09-19 | 0 | 0 | 0 | 0 | 0 |
| Klebsormidium nitens | GCA_000708835.1_ASM70883v1 | 104,21 | 2014-05-29 | 0 | 0 | 0 | 0 | 0 |
| Kokia drynarioides | GCA_002814295.1_KokDry1 | 517,43 | 2017-12-08 | 0 | 0 | 0 | 0 | 0 |
| Lactuca sativa | GCA_000227445.1_Legassy_V2 | 1133,66 | 2011-10-04 | 0 | 0 | 0 | 0 | 0 |
| Lactuca sativa | GCA_900198505.1_Lsativa | 1975,25 | 2017-09-07 | 0 | 0 | 0 | 21 | 0 |
| Lactuca sativa | GCA_900243165.1_Salat_v21 | 2224,43 | 2017-12-20 | 1 | 0 | 0 | 646 | 0 |
| Lactuca sativa | GCA_002870075.1_Lsat_Salinas_v7 | 2384,19 | 2018-01-09 | 0 | 0 | 0 | 0 | 0 |
| Lagenaria siceraria | GCA_000466325.1_Bottle_gourd | 176,727 | 2013-09-11 | 0 | 0 | 1 | 148 | 0 |
| Lagenaria siceraria | GCA_002890555.1_BG_Scaf | 326,59 | 2018-01-17 | 0 | 0 | 0 | 2 | 0 |
| Lagenaria siceraria | GCA_003268545.1_Lsi_v1.0 | 313,387 | 2018-06-27 | 0 | 0 | 0 | 0 | 0 |
| Leavenworthia alabamica | GCA_000411055.1_VEGI_LA_v_1.0 | 173,432 | 2013-06-12 | 0 | 0 | 0 | 0 | 0 |
| Leersia perrieri | GCA_000325765.3_Lperr_V1.4 | 266,688 | 2012-10-17 | 0 | 0 | 0 | 0 | 0 |
| Linum usitatissimum | GCA_000224295.2_ASM22429v2 | 316,167 | 2018-03-09 | 0 | 0 | 0 | 0 | 0 |
| Liriodendron chinense | GCA_003013855.2_NJFU_Lchi_2.0 | 1742,42 | 2018-03-21 | 0 | 0 | 0 | 0 | 0 |
| Lolium perenne | GCA_001735685.1_ASM173568v1 | 481,479 | 2016-09-20 | 20 | 0 | 0 | 103 | 0 |
| Lophocereus schottii | GCA_002740545.1_Lsch_v1.3 | 797,926 | 2017-10-31 | 0 | 0 | 0 | 0 | 0 |
| Lotus japonicus | GCA_000181115.2_Lj3.0 | 394,455 | 2008-05-28 | 4 | 0 | 1 | 7 | 0 |
| Lupinus angustifolius | GCA_000338175.1_Lupin_genome_scaffold | 523,298 | 2013-02-06 | 160 | 0 | 2 | 18 | 0 |
| Lupinus angustifolius | GCA_001865875.1_LupAngTanjil_v1.0 | 609,203 | 2016-11-01 | 32 | 0 | 0 | 2 | 0 |
| Lupinus angustifolius | GCA_002285895.2_ASM228589v2 | 557,909 | 2017-09-05 | 5 | 0 | 0 | 2 | 0 |
| Macadamia integrifolia | GCA_900087525.1_Macadmia_integrifolia_v1.1 | 518,49 | 2016-08-22 | 0 | 0 | 0 | 0 | 0 |
| Macleaya cordata | GCA_002174775.1_MC_HNAU_1.0 | 377,834 | 2017-06-07 | 0 | 0 | 0 | 0 | 0 |
| Magnolia ashei | GCA_003571905.1_ASM357190v1 | 284,512 | 2018-09-17 | 0 | 0 | 0 | 0 | 0 |
| Malus domestica | GCA_000148765.2_MalDomGD1.0 | 1874,77 | 2010-09-03 | 0 | 0 | 0 | 0 | 0 |
| Malus domestica | GCA_002114115.1_ASM211411v1 | 702,961 | 2017-04-28 | 3 | 0 | 0 | 0 | 0 |
| Manihot esculenta | GCA_000737115.1_MK_v2b | 292,098 | 2014-07-31 | 1 | 0 | 0 | 0 | 0 |
| Manihot esculenta | GCA_001659605.1_Manihot_esculenta_v6 | 582,279 | 2016-06-07 | 0 | 0 | 0 | 0 | 0 |
| Manihot esculenta subsp. flabellifolia | GCA_000737105.1_MW_v2d | 390,836 | 2014-07-31 | 0 | 0 | 0 | 1 | 0 |
| Marchantia polymorpha | GCA_003032435.1_Marchanta_polymorpha_v1 | 225,761 | 2018-01-10 | 0 | 0 | 0 | 0 | 0 |
| Marchantia polymorpha subsp. ruderalis | GCA_001641455.1_Mp_v4 | 205,718 | 2016-05-10 | 0 | 0 | 1 | 262 | 0 |
| Medicago truncatula | GCA_000219495.2_MedtrA17_4.0 | 412,924 | 2011-08-12 | 2 | 0 | 0 | 244 | 0 |
| Medicago truncatula | GCA_002024945.1_R108_v._1.0 | 402,065 | 2017-03-13 | 8 | 0 | 0 | 4 | 0 |
| Medicago truncatula | GCA_002251925.1_hm034.alpaca.scaffolds | 427,447 | 2017-08-15 | 0 | 0 | 0 | 0 | 0 |
| Medicago truncatula | GCA_002251935.1_hm340.alpaca.scaffolds | 394,059 | 2017-08-15 | 0 | 0 | 0 | 2 | 0 |
| Medicago truncatula | GCA_002251955.1_hm056.alpaca.scaffolds | 426,024 | 2017-08-15 | 0 | 0 | 0 | 0 | 0 |
| Medicago truncatula | GCA_003473485.2_MtrunA17r5.0-ANR | 429,612 | 2018-09-06 | 6 | 0 | 0 | 0 | 0 |
| Mentha longifolia | GCA_001642375.1_Mlong1.0 | 353,287 | 2016-05-11 | 0 | 0 | 0 | 4 | 0 |
| Metrosideros polymorpha var. glaberrima | GCA_001662345.1_Mpo_1.0 | 304,366 | 2016-04-28 | 36 | 0 | 0 | 10 | 0 |
| Micractinium conductrix | GCA_002245815.2_ASM224581v2 | 61,0189 | 2017-08-10 | 0 | 0 | 0 | 0 | 0 |
| Micromonas commoda | GCA_000090985.2_ASM9098v2 | 21,1093 | 2009-04-10 | 0 | 0 | 0 | 0 | 0 |
| Micromonas pusilla CCMP1545 | GCA_000151265.1_Micromonas_pusilla_CCMP1545_v2.0 | 21,9583 | 2009-04-07 | 0 | 0 | 0 | 0 | 0 |
| Micromonas sp. ASP10-01a | GCA_001430725.1_ASM143072v1 | 19,5824 | 2015-04-28 | 0 | 0 | 0 | 0 | 0 |
| Mimosa pudica | GCA_003254945.1_ASM325494v1 | 557,202 | 2018-06-19 | 5 | 0 | 0 | 0 | 0 |
| Miscanthus sacchariflorus | GCA_002993905.1_Msac_v3 | 2074,92 | 2018-03-09 | 1 | 0 | 0 | 0 | 0 |
| Momordica charantia | GCA_001995035.1_ASM199503v1 | 285,614 | 2016-12-26T20:44:00Z | 0 | 0 | 0 | 0 | 0 |
| Momordica charantia | GCA_900491585.1_M._spontanea_TR | 296,263 | 2018-06-22 | 3 | 0 | 0 | 0 | 0 |
| Monoraphidium neglectum | GCA_000611645.1_mono_v1 | 69,7118 | 2014-03-28 | 0 | 0 | 0 | 0 | 0 |
| Monoraphidium sp. 549 | GCA_002814315.1_ASM281431v1 | 74,6589 | 2017-12-08 | 0 | 0 | 0 | 3 | 0 |
| Monotropa hypopitys | GCA_002855965.1_monotropa1.0 | 2197,49 | 2018-01-03 | 1 | 0 | 0 | 0 | 0 |
| Morus notabilis | GCA_000414095.2_ASM41409v2 | 320,379 | 2013-06-24 | 15 | 0 | 0 | 63 | 0 |
| Mucuna pruriens | GCA_003370565.1_ASM337056v1 | 397,042 | 2018-08-10 | 0 | 0 | 0 | 0 | 0 |
| Musa acuminata subsp. malaccensis | GCA_000313855.2_ASM31385v2 | 472,231 | 2012-08-09 | 0 | 0 | 0 | 0 | 0 |
| Musa itinerans | GCA_001649415.1_ASM164941v1 | 455,349 | 2016-05-21 | 0 | 0 | 0 | 5 | 0 |
| Nelumbo nucifera | GCA_000365185.2_Chinese_Lotus_1.1 | 804,648 | 2013-04-15 | 0 | 0 | 0 | 1 | 0 |
| Nelumbo nucifera | GCA_000805495.1_Nelumbo_nucifera_v1.1 | 790,339 | 2014-12-19 | 0 | 0 | 0 | 0 | 0 |
| Nelumbo nucifera | GCA_003033685.1_ASM303368v1 | 817,268 | 2018-04-04 | 0 | 0 | 0 | 1 | 0 |
| Nelumbo nucifera | GCA_003033695.1_ASM303369v1 | 799,479 | 2018-04-04 | 0 | 0 | 0 | 0 | 0 |
| Nicotiana attenuata | GCA_001879085.1_NIATTr2 | 2365,68 | 2016-11-14 | 21 | 0 | 0 | 18 | 0 |
| Nicotiana attenuata | GCA_002018495.1_NIATT_ARIZONA | 1827,78 | 2017-03-06 | 4 | 0 | 0 | 0 | 0 |
| Nicotiana benthamiana | GCA_000723945.1_Ni_ben | 61,9511 | 2014-05-27 | 0 | 0 | 0 | 5 | 0 |
| Nicotiana glauca | GCA_002930595.1_NicGla1.0 | 3222,83 | 2018-02-16 | 5 | 0 | 0 | 68 | 0 |
| Nicotiana obtusifolia | GCA_002018475.1_NIOBT.version3 | 1222,77 | 2017-03-06 | 1 | 0 | 0 | 3 | 0 |
| Nicotiana otophora | GCA_000715115.1_Noto | 2689,35 | 2014-06-05 | 62 | 0 | 1 | 10 | 0 |
| Nicotiana sylvestris | GCA_000393655.1_Nsyl | 2221,99 | 2013-05-08 | 25 | 0 | 0 | 6 | 0 |
| Nicotiana tabacum | GCA_000715075.1_Ntab-K326 | 3732,64 | 2014-05-29 | 43 | 0 | 0 | 8 | 0 |
| Nicotiana tabacum | GCA_000715095.1_Ntab-BX | 3735,82 | 2014-05-29 | 41 | 0 | 0 | 8 | 0 |
| Nicotiana tabacum | GCA_000715135.1_Ntab-TN90 | 3643,47 | 2014-05-29 | 30 | 0 | 0 | 4 | 0 |
| Nicotiana tabacum | GCA_002210045.1_Nitab4.5 | 4646,65 | 2017-06-28 | 23 | 0 | 0 | 10 | 0 |
| Nicotiana tomentosiformis | GCA_000390325.2_Ntom_v01 | 1688,47 | 2013-05-10 | 6 | 0 | 0 | 6 | 0 |
| Nissolia schottii | GCA_003254905.1_ASM325490v1 | 466,099 | 2018-06-19 | 0 | 0 | 0 | 2 | 0 |
| Nothapodytes nimmoniana | GCA_002091855.1_Nnimmo_assembly01 | 1,36527 | 2017-04-03T20:46:00Z | 0 | 0 | 0 | 0 | 0 |
| Ochetophila trinervis | GCA_003254975.1_ASM325497v1 | 309,116 | 2018-06-19 | 0 | 0 | 0 | 0 | 0 |
| Ocimum tenuiflorum | GCA_001278415.1_OciTen1.0 | 332,617 | 2015-09-02 | 1 | 0 | 0 | 6 | 0 |
| Ocimum tenuiflorum | GCA_001748785.1_OcimTenu2.0 | 311,125 | 2016-09-29 | 2 | 0 | 0 | 7 | 0 |
| Olea europaea var. sylvestris | GCA_002742605.1_O_europaea_v1 | 1141,15 | 2017-11-01 | 0 | 0 | 0 | 1 | 0 |
| Oropetium thomaeum | GCA_001182835.1_Oropetium_genomic_20141112 | 243,175 | 2015-07-20 | 0 | 0 | 0 | 0 | 0 |
| Oryza barthii | GCA_000182155.3_O.barthii_v1.3 | 308,272 | 2009-04-21 | 0 | 0 | 0 | 0 | 0 |
| Oryza barthii | GCA_002926215.1_LB120v1 | 295,586 | 2018-02-13 | 1 | 0 | 0 | 0 | 0 |
| Oryza barthii | GCA_002926235.1_TB65v1 | 294,191 | 2018-02-13 | 1 | 0 | 0 | 0 | 0 |
| Oryza barthii | GCA_003020155.1_B88v1 | 292,235 | 2018-03-27 | 0 | 0 | 0 | 0 | 0 |
| Oryza brachyantha | GCA_000231095.2_Oryza_brachyantha.v1.4b | 259,908 | 2011-10-19 | 0 | 0 | 0 | 0 | 0 |
| Oryza brachyantha | GCA_000710545.1_O._brachyantha_chromosome_3_short_arm | 14,4404 | 2014-06-20 | 0 | 0 | 0 | 0 | 0 |
| Oryza glaberrima | GCA_000147395.2_Oryza_glaberrima_V1 | 303,295 | 2010-07-08 | 0 | 0 | 0 | 2 | 0 |
| Oryza glumipatula | GCA_000576495.1_Oryza_glumaepatula_v1.5 | 372,86 | 2013-02-06 | 0 | 0 | 0 | 0 | 0 |
| Oryza longistaminata | GCA_000789195.1_O_longistaminata_v1.0 | 326,443 | 2014-12-03 | 1 | 0 | 0 | 1 | 0 |
| Oryza longistaminata | GCA_001514335.2_ASM151433v2 | 362,064 | 2016-01-13 | 0 | 0 | 0 | 1 | 0 |
| Oryza meridionalis | GCA_000338895.2_Oryza_meridionalis_v1.3 | 335,668 | 2012-10-17 | 0 | 0 | 0 | 0 | 0 |
| Oryza meridionalis | GCA_001551795.1_ASM155179v1 | 354,611 | 2016-02-07 | 0 | 0 | 0 | 0 | 0 |
| Oryza meyeriana var. granulata | GCA_000325645.2_OgranChr3sV1 | 35,2457 | 2012-10-17 | 0 | 0 | 0 | 0 | 0 |
| Oryza minuta | GCA_000632695.1_ASM63269v1 | 45,1659 | 2014-04-16 | 0 | 0 | 0 | 0 | 0 |
| Oryza officinalis | GCA_000717455.1_O._officinalis_chromosome_3_short_arm | 26,1885 | 2014-07-02 | 0 | 0 | 0 | 0 | 0 |
| Oryza punctata | GCA_000573905.1_Oryza_punctata_v1.2 | 393,817 | 2014-02-19 | 0 | 0 | 0 | 0 | 0 |
| Oryza punctata | GCA_000710525.1_O._punctata_chromosome_3_short_arm | 22,4654 | 2014-06-20 | 0 | 0 | 0 | 0 | 0 |
| Oryza rufipogon | GCA_000700045.1_O._rufipogon_chromosome_3_short_arm | 12,7409 | 2014-06-10 | 0 | 0 | 0 | 0 | 0 |
| Oryza rufipogon | GCA_000817225.1_OR_W1943 | 339,177 | 2014-12-30 | 1 | 0 | 0 | 2 | 0 |
| Oryza rufipogon | GCA_001551805.1_ASM155180v1 | 384,518 | 2016-02-07 | 2 | 0 | 0 | 0 | 0 |
| Oryza sativa | GCA_001648735.1_ASM164873v1 | 307,225 | 2016-05-19 | 0 | 0 | 0 | 0 | 0 |
| Oryza sativa | GCA_001648745.1_IL50-13 | 295,39 | 2016-05-19 | 3 | 0 | 0 | 0 | 0 |
| Oryza sativa aus subgroup | GCA_001952365.1_ASM195236v1 | 362,279 | 2017-01-11 | 1 | 0 | 0 | 0 | 0 |
| Oryza sativa f. spontanea | GCA_000576065.1_Oryza_nivara_v1.0 | 337,95 | 2014-02-19 | 0 | 0 | 0 | 0 | 0 |
| Oryza sativa f. spontanea | GCA_000710535.2_ASM71053v2 | 19,4244 | 2014-06-20 | 0 | 0 | 0 | 0 | 0 |
| Oryza sativa Indica Group | GCA_000004655.2_ASM465v1 | 426,337 | 2002-04-04 | 2 | 0 | 0 | 1703 | 0 |
| Oryza sativa Indica Group | GCA_000725085.2_ASM72508v2 | 389,753 | 2014-07-11 | 0 | 0 | 0 | 1 | 0 |
| Oryza sativa Indica Group | GCA_001305255.1_ASM130525v1 | 352,121 | 2015-10-01 | 0 | 0 | 0 | 0 | 0 |
| Oryza sativa Indica Group | GCA_001611195.1_ASM161119v1 | 351,225 | 2016-04-05 | 3 | 0 | 0 | 1 | 0 |
| Oryza sativa Indica Group | GCA_001611235.1_ASM161123v1 | 352,227 | 2016-04-05 | 30 | 0 | 0 | 6 | 0 |
| Oryza sativa Indica Group | GCA_001611255.1_ASM161125v1 | 331,819 | 2016-04-05 | 13 | 0 | 0 | 5 | 0 |
| Oryza sativa Indica Group | GCA_001618785.1_Mhv2.0 | 398,762 | 2016-04-11 | 0 | 0 | 0 | 0 | 0 |
| Oryza sativa Indica Group | GCA_001618795.1_ZSv2.0 | 386,486 | 2016-04-11 | 0 | 0 | 0 | 0 | 0 |
| Oryza sativa Indica Group | GCA_001623345.2_ZS97RS2 | 387,326 | 2016-04-18 | 11 | 0 | 0 | 0 | 0 |
| Oryza sativa Indica Group | GCA_001623365.2_MH63RS2 | 387,424 | 2016-04-18 | 3 | 0 | 0 | 0 | 0 |
| Oryza sativa Indica Group | GCA_001889745.1_Rice_IR8_v1.7 | 389,088 | 2016-12-06 | 6 | 0 | 0 | 0 | 0 |
| Oryza sativa Indica Group | GCA_002151415.1_R498.Genome.version1 | 390,984 | 2017-05-04 | 11 | 0 | 0 | 0 | 0 |
| Oryza sativa Indica Group | GCA_003449045.1_ASM344904v1 | 388,772 | 2018-09-04 | 0 | 0 | 0 | 0 | 0 |
| Oryza sativa Japonica Group | GCA_000149285.1_OrySat_Sep2003 | 391,148 | 2004-10-21 | 0 | 0 | 0 | 1126 | 0 |
| Oryza sativa Japonica Group | GCA_000005425.2_Build_4.0 | 382,778 | 2005-02-02 | 0 | 0 | 0 | 0 | 0 |
| Oryza sativa Japonica Group | GCA_000164945.1_OrySat_Aug2009 | 382,151 | 2010-04-01 | 0 | 0 | 0 | 0 | 0 |
| Oryza sativa Japonica Group | GCA_000321445.1_Osat_hitom_01 | 382,627 | 2011-12-27 | 0 | 0 | 0 | 0 | 0 |
| Oryza sativa Japonica Group | GCA_000817615.1_HEG4v1.0 | 342,028 | 2015-01-12 | 0 | 0 | 0 | 0 | 0 |
| Oryza sativa Japonica Group | GCA_000817635.1_A123v1.0 | 337,74 | 2015-01-12 | 0 | 0 | 0 | 0 | 0 |
| Oryza sativa Japonica Group | GCA_001433935.1_IRGSP-1.0 | 374,423 | 2015-10-10 | 0 | 0 | 0 | 0 | 0 |
| Oryza sativa Japonica Group | GCA_002573525.1_SJ18_v1 | 418,901 | 2017-10-17 | 5 | 0 | 0 | 718 | 0 |
| Oryza sativa Japonica Group | GCA_003449065.1_ASM344906v1 | 378,097 | 2018-09-04 | 0 | 0 | 0 | 0 | 0 |
| Ostreococcus lucimarinus CCE9901 | GCA_000092065.1_ASM9206v1 | 13,2049 | 2007-04-10 | 0 | 0 | 0 | 0 | 0 |
| Ostreococcus tauri | GCA_000214015.2_version_140606 | 13,0328 | 2010-10-15 | 0 | 0 | 0 | 0 | 0 |
| Ostreococcus tauri | GCA_002158475.1_Ostta1115_2 | 14,7627 | 2017-05-26 | 0 | 0 | 0 | 0 | 0 |
| Pachycereus pringlei | GCA_002740445.1_Ppri_v1.3 | 629,656 | 2017-10-31 | 0 | 0 | 0 | 0 | 0 |
| Panicum hallii | GCA_002211085.2_PHallii_v3.1 | 535,889 | 2017-06-19 | 9 | 0 | 0 | 2 | 0 |
| Panicum hallii var. hallii | GCA_003061485.1_PhalliiHAL_v2.1 | 487,474 | 2018-04-23 | 7 | 0 | 0 | 0 | 0 |
| Panicum miliaceum | GCA_002895445.2_ASM289544v2 | 848,352 | 2018-01-23 | 0 | 0 | 0 | 0 | 0 |
| Panicum miliaceum | GCA_003046395.2_Pm_0390_v2 | 854,793 | 2018-04-09 | 13 | 0 | 0 | 1 | 0 |
| Papaver somniferum | GCA_003573695.1_ASM357369v1 | 2715,53 | 2018-09-10 | 3 | 0 | 0 | 1 | 0 |
| Parachlorella kessleri | GCA_001598975.1_PK2152_assembly | 59,1878 | 2015-12-19 | 0 | 0 | 0 | 0 | 0 |
| Parasponia andersonii | GCA_002914805.1_PanWU01x14_asm01 | 475,834 | 2018-02-02 | 0 | 0 | 0 | 0 | 0 |
| Passiflora edulis | GCA_002156105.1_ASM215610v1 | 165,657 | 2017-05-22 | 0 | 0 | 0 | 0 | 0 |
| Penstemon barbatus | GCA_003313485.1_Duke_Pbarb_2016 | 696,306 | 2018-07-11 | 2 | 0 | 0 | 0 | 0 |
| Penstemon centranthifolius | GCA_000737435.1_ASM73743v1 | 4,47159 | 2014-08-04 | 0 | 0 | 0 | 0 | 0 |
| Penstemon cyananthus | GCA_000281005.1_ASM28100v1 | 4,62226 | 2012-07-23 | 0 | 0 | 0 | 1 | 0 |
| Penstemon davidsonii | GCA_000280985.1_ASM28098v1 | 2,37523 | 2012-07-23 | 0 | 0 | 0 | 1 | 0 |
| Penstemon dissectus | GCA_000280965.1_ASM28096v1 | 2,62809 | 2012-07-23 | 1 | 0 | 0 | 1 | 0 |
| Penstemon fruticosus | GCA_000281025.1_ASM28102v1 | 2,31904 | 2012-07-23 | 0 | 0 | 0 | 0 | 0 |
| Penstemon grinnellii | GCA_000737425.1_ASM73742v1 | 3,66352 | 2014-08-04 | 0 | 0 | 0 | 0 | 0 |
| Pereskia humboldtii | GCA_002740485.1_Phum_v1.3 | 414,047 | 2017-10-31 | 0 | 0 | 0 | 0 | 0 |
| Persea americana | GCA_002908915.1_Hass1.0 | 446,756 | 2018-01-30 | 0 | 0 | 0 | 0 | 0 |
| Phalaenopsis aphrodite | GCA_003013225.1_ASM301322v1 | 1025,1 | 2018-03-21 | 0 | 0 | 0 | 0 | 0 |
| Phalaenopsis equestris | GCA_001263595.1_ASM126359v1 | 1064,2 | 2015-08-07 | 3 | 0 | 1 | 1 | 1 |
| Phalaenopsis hybrid cultivar | GCA_002079205.1_ASM207920v1 | 2687,66 | 2017-04-04 | 0 | 0 | 0 | 1 | 0 |
| Phaseolus coccineus subsp. coccineus | GCA_003122825.1_UCLA_Phcoc_1.0 | 371,086 | 2018-05-14 | 2 | 0 | 0 | 6 | 0 |
| Phaseolus vulgaris | GCA_000499845.1_PhaVulg1_0 | 521,077 | 2013-11-26 | 0 | 0 | 0 | 0 | 0 |
| Phaseolus vulgaris | GCA_001517995.1_phasIbeam10.0 | 549,748 | 2016-01-15 | 0 | 0 | 0 | 333 | 0 |
| Phoenix dactylifera | GCA_000181215.2_PDK_30 | 381,563 | 2009-10-09 | 0 | 0 | 0 | 0 | 0 |
| Phoenix dactylifera | GCA_000413155.1_DPV01 | 556,481 | 2013-06-19 | 3 | 0 | 0 | 84 | 0 |
| Physcomitrella patens | GCA_000002425.2_Phypa_V3 | 472,081 | 2007-11-27 | 0 | 0 | 0 | 0 | 0 |
| Picea glauca | GCA_000411955.5_PG29_v4.1 | 24633,1 | 2013-05-17 | 0 | 0 | 0 | 0 | 0 |
| Picea glauca | GCA_000966675.1_WS77111_V1 | 26936,2 | 2015-03-13 | 1 | 0 | 0 | 24 | 3 |
| Picea glauca | GCA_001687225.1_SeqCapPg29 | 258,272 | 2016-07-18 | 2 | 0 | 0 | 0 | 0 |
| Picochlorum sp. 'soloecismus' | GCA_002818215.1_ASM281821v1 | 15,2525 | 2017-12-08 | 0 | 0 | 0 | 0 | 0 |
| Picochlorum sp. SENEW3 | GCA_000876415.1_ASM87641v1 | 13,3907 | 2014-07-28 | 0 | 0 | 0 | 0 | 0 |
| Picocystis sp. ML | GCA_003665715.1_OU_Pico_1.0 | 29,6462 | 2018-10-16 | 0 | 0 | 0 | 0 | 0 |
| Pinus lambertiana | GCA_001447015.2_Sugar_pine_JHU_assembly | 27602,7 | 2015-11-17 | 1323 | 0 | 0 | 624 | 0 |
| Pinus sylvestris | GCA_900143225.1_FinPunk_mtDNA_Assembly | 0,985624 | 2017-01-02 | 0 | 0 | 0 | 0 | 0 |
| Pinus taeda | GCA_000404065.3_Ptaeda2.0 | 22103,6 | 2015-08-06 | 13 | 0 | 0 | 62 | 0 |
| Pisum sativum | GCA_003013575.1_ASM301357v1 | 4275,93 | 2018-03-21 | 51842 | 0 | 405 | 23546 | 298 |
| Pogostemon cablin | GCA_003675935.1_DSBC_Pcab_1.0 | 1916,69 | 2018-10-23 | 0 | 0 | 0 | 70 | 0 |
| Populus euphratica | GCA_000495115.1_PopEup_1.0 | 496,033 | 2013-11-01 | 3 | 0 | 2 | 26 | 0 |
| Populus trichocarpa | GCA_000002775.3_Pop_tri_v3 | 434,29 | 2006-09-14 | 0 | 0 | 0 | 0 | 0 |
| Primula veris | GCA_000788445.1_ASM78844v1 | 309,693 | 2014-12-03 | 1 | 0 | 0 | 1 | 0 |
| Primula vulgaris | GCA_001077355.1_ASM107735v1 | 1,50478 | 2015-01-28 | 0 | 0 | 0 | 3 | 0 |
| Primula vulgaris | GCA_001403715.1_Primula_Slocus | 1,50478 | 2015-09-19 | 0 | 0 | 0 | 3 | 0 |
| Prototheca cutis | GCA_002897115.1_JCM_15793_assembly_v001 | 19,9692 | 2017-11-16 | 0 | 0 | 0 | 0 | 0 |
| Prototheca stagnorum | GCA_002794665.1_JCM_9641_assembly_v001 | 16,8962 | 2017-11-16 | 0 | 0 | 0 | 0 | 0 |
| Prototheca wickerhamii | GCA_003255715.1_ASM325571v1 | 27,6911 | 2018-05-11 | 0 | 0 | 0 | 0 | 0 |
| Prototheca zopfii | GCA_003612995.1_ASM361299v1 | 24,7449 | 2018-10-04 | 0 | 0 | 0 | 2 | 0 |
| Prototheca zopfii | GCA_003613005.1_ASM361300v1 | 26,4489 | 2018-10-04 | 0 | 0 | 0 | 7 | 0 |
| Prunus avium | GCA_002207925.1_PAV_r1.0 | 272,362 | 2017-06-01 | 0 | 0 | 0 | 0 | 0 |
| Prunus mume | GCA_000346735.1_P.mume_V1.0 | 234,03 | 2013-02-28 | 0 | 0 | 0 | 3 | 0 |
| Prunus persica | GCA_000218175.1_PrunusPersicaDD_1.0 | 214,225 | 2011-06-01 | 8 | 0 | 0 | 3 | 0 |
| Prunus persica | GCA_000218195.1_PrunusPersicaGB_1.0 | 211,308 | 2011-06-01 | 2 | 0 | 0 | 0 | 0 |
| Prunus persica | GCA_000218215.1_PrunusPersicaF8_1.0 | 207,185 | 2011-06-01 | 9 | 0 | 0 | 0 | 0 |
| Prunus persica | GCA_000346465.2_Prunus_persica_NCBIv2 | 227,569 | 2013-03-14 | 0 | 0 | 0 | 0 | 0 |
| Prunus yedoensis var. nudiflora | GCA_002966975.1_Pyn_1.0 | 85,0419 | 2018-03-02 | 0 | 0 | 0 | 0 | 0 |
| Prunus yedoensis var. nudiflora | GCA_900382725.1_Pyn.v1 | 319,21 | 2018-07-08 | 0 | 0 | 0 | 0 | 0 |
| Pseudotsuga menziesii | GCA_001517045.1_DougFir1.0 | 14673,2 | 2015-12-22 | 2662 | 0 | 0 | 1092 | 0 |
| Psidium guajava | GCA_002914565.1_Guava1.0 | 386,852 | 2018-02-02 | 2 | 0 | 0 | 0 | 0 |
| Pterocarya stenoptera | GCA_003123785.1_ASM312378v1 | 955,601 | 2018-05-14 | 33 | 0 | 0 | 126 | 0 |
| Punica granatum | GCA_002201585.1_ASM220158v1 | 296,383 | 2017-06-20 | 0 | 0 | 0 | 1 | 0 |
| Punica granatum | GCA_002837095.1_ASM283709v1 | 380,178 | 2017-12-12 | 1 | 0 | 0 | 0 | 0 |
| Punica granatum | GCA_002864125.1_ASM286412v1 | 274,043 | 2018-01-05 | 0 | 0 | 0 | 3 | 0 |
| Purshia tridentata | GCA_003254885.1_ASM325488v1 | 175,971 | 2018-06-19 | 0 | 0 | 0 | 0 | 0 |
| Pyrus x bretschneideri | GCA_000315295.1_Pbr_v1.0 | 508,551 | 2012-11-21 | 0 | 0 | 0 | 0 | 0 |
| Quercus lobata | GCA_001633185.1_ValleyOak0.5 | 759,241 | 2016-04-28 | 0 | 0 | 0 | 0 | 0 |
| Quercus robur | GCA_900291515.1_Q_robur_v1 | 814,336 | 2018-03-09 | 0 | 0 | 0 | 0 | 0 |
| Quercus robur | GCA_003013145.1_ASM301314v1 | 719,602 | 2018-03-21 | 0 | 0 | 0 | 0 | 0 |
| Quercus suber | GCA_002906115.1_CorkOak1.0 | 953,299 | 2018-01-29T11:22:00Z | 3 | 0 | 0 | 1 | 0 |
| Quillaja saponaria | GCA_003338715.1_Draft.Quillaja.v1.0 | 248,908 | 2018-07-24 | 0 | 0 | 0 | 0 | 0 |
| Raphanus raphanistrum subsp. raphanistrum | GCA_000769845.1_ASM76984v1 | 253,834 | 2014-10-28 | 0 | 0 | 0 | 2 | 0 |
| Raphanus sativus | GCA_000715565.1_RSA_r1.0 | 402,328 | 2014-06-05 | 0 | 0 | 0 | 0 | 0 |
| Raphanus sativus | GCA_000801105.2_Rs1.0 | 426,614 | 2014-12-12 | 0 | 0 | 0 | 0 | 0 |
| Raphanus sativus | GCA_001047155.1_Rsativus_build1.00 | 383,105 | 2015-06-09 | 589 | 0 | 0 | 40 | 0 |
| Raphanus sativus | GCA_002197605.1_Radish_v2.20 | 382,79 | 2017-06-15 | 3 | 0 | 0 | 16 | 0 |
| Raphidocelis subcapitata | GCA_003203535.1_Rsub_1.0 | 51,1627 | 2018-05-30 | 0 | 0 | 0 | 0 | 0 |
| Rhazya stricta | GCA_001752375.1_RHA1.0 | 274,354 | 2016-10-04 | 0 | 0 | 0 | 0 | 0 |
| Rhizophora apiculata | GCA_900004065.1_ra_scaffold | 232,431 | 2017-09-19 | 4 | 0 | 0 | 373 | 0 |
| Rhizophora apiculata | GCA_900174605.1_Rap_scaffold_v2 | 232,055 | 2017-09-19 | 19 | 0 | 0 | 2 | 0 |
| Ricinus communis | GCA_000151685.2_JCVI_RCG_1.1 | 350,622 | 2006-07-18 | 5 | 0 | 0 | 445 | 0 |
| Rosa chinensis | GCA_002994745.1_RchiOBHm-V2 | 513,854 | 2018-03-13 | 0 | 0 | 0 | 0 | 0 |
| Rosa multiflora | GCA_002564525.1_RMU_r2.0 | 739,638 | 2017-10-12 | 0 | 0 | 0 | 0 | 0 |
| Rosa x damascena | GCA_001662545.1_ASM166254v1 | 711,72 | 2016-06-13 | 12 | 0 | 0 | 0 | 0 |
| Ruellia speciosa | GCA_001909325.1_Rspec1.0 | 740,036 | 2016-12-20 | 0 | 0 | 0 | 0 | 0 |
| Saccharum hybrid cultivar | GCA_900465005.1_MTP | 530,66 | 2018-06-18 | 0 | 0 | 0 | 210 | 0 |
| Saccharum hybrid cultivar SP80-3280 | GCA_002018215.1_CTBE_SP803280_v1.0 | 1169,95 | 2017-03-03 | 0 | 0 | 0 | 0 | 0 |
| Saccharum spontaneum | GCA_900500655.1_Sugarcane_genome | 3924,19 | 2018-08-07 | 1925 | 0 | 6 | 36 | 3 |
| Saccharum spontaneum | GCA_003544955.1_Sspon.HiC_chr_asm | 3133,29 | 2018-09-10 | 21 | 0 | 0 | 1203 | 0 |
| Santalum album | GCA_002911635.1_ASM291163v1 | 196,101 | 2018-02-01 | 0 | 0 | 0 | 0 | 0 |
| Santalum album | GCA_002925775.1_SaGenomev1.0 | 220,961 | 2018-02-12 | 1 | 0 | 0 | 13 | 0 |
| Scenedesmus quadricauda | GCA_002317545.1_ASM231754v1 | 65,3539 | 2017-09-20 | 0 | 0 | 0 | 2 | 0 |
| Schrenkiella parvula | GCA_000218505.1_Eutrema_parvulum_v01 | 137,073 | 2011-03-25 | 0 | 0 | 0 | 0 | 0 |
| Secale cereale | GCA_900002355.1_Rye_Select_Lo7_assembly | 1684,93 | 2016-11-23 | 0 | 0 | 0 | 0 | 0 |
| Secale cereale | GCA_900079665.1_Rye_Lo7_WGS_contigs | 1684,93 | 2016-11-23 | 0 | 0 | 0 | 0 | 0 |
| Selaginella kraussiana | GCA_001021135.1_ASM102113v1 | 114,503 | 2015-06-05 | 0 | 0 | 0 | 3 | 0 |
| Selaginella moellendorffii | GCA_000143415.2_v1.0 | 212,315 | 2010-06-24 | 0 | 0 | 0 | 1 | 0 |
| Selaginella tamariscina | GCA_003024785.1_ASM302478v1 | 300,729 | 2018-03-29 | 0 | 0 | 0 | 0 | 0 |
| Sesamum indicum | GCA_000512975.1_S_indicum_v1.0 | 275,059 | 2013-12-24 | 0 | 0 | 0 | 0 | 0 |
| Sesamum indicum | GCA_000975565.1_ASM97556v1 | 340,464 | 2015-04-22 | 0 | 0 | 0 | 0 | 0 |
| Sesamum indicum | GCA_001692995.1_S_indicum_Yuzhi11_v1 | 210,758 | 2016-07-24 | 0 | 0 | 0 | 635 | 0 |
| Sesamum indicum | GCA_003268515.1_ASM326851v1 | 242,679 | 2018-06-26 | 1 | 0 | 0 | 2 | 0 |
| Setaria italica | GCA_000263155.2_Setaria_italica_v2.0 | 405,868 | 2012-05-11 | 0 | 0 | 0 | 0 | 0 |
| Setaria italica | GCA_001652605.1_ASM165260v1 | 477,542 | 2016-05-26 | 0 | 0 | 0 | 0 | 0 |
| Silene latifolia | GCA_900095335.1_trinity_orf_cap3 | 36,0486 | 2016-11-03 | 0 | 0 | 1 | 888 | 0 |
| Silene latifolia | GCA_003260165.1_S_latifolia_v1.0 | 1185,09 | 2018-06-22 | 2 | 0 | 0 | 0 | 0 |
| Silene latifolia subsp. alba | GCA_001412135.1_ASM141213v1 | 665,279 | 2015-10-23 | 2 | 0 | 0 | 5 | 0 |
| Silybum marianum | GCA_001541825.1_ASM154182v1 | 1477,57 | 2016-01-26 | 4 | 0 | 0 | 14 | 0 |
| Sisymbrium irio | GCA_000411075.1_VEGI_SI_v_1.0 | 245,55 | 2013-06-13 | 0 | 0 | 0 | 0 | 0 |
| Solanum americanum | GCA_900188785.1_NanoPore_RenSeq_-_Canu_nanopolish | 7,74921 | 2017-07-21 | 0 | 0 | 0 | 0 | 0 |
| Solanum americanum | GCA_900188835.1_S._americanum_SMRT_RenSeq_PacBio_Canu | 9,83576 | 2017-07-21 | 0 | 0 | 0 | 0 | 0 |
| Solanum americanum | GCA_900188885.1_Nanopore_RenSeq_Canu_assembly | 7,6066 | 2017-07-21 | 0 | 0 | 0 | 0 | 0 |
| Solanum americanum | GCA_900188895.1_NanoPore_RenSeq_-_Canu_assembly_nanopolish_pilon | 7,74473 | 2017-07-21 | 0 | 0 | 0 | 0 | 0 |
| Solanum americanum | GCA_900188915.1_S._americanum_SMRT_RenSeq_Geneious_R8 | 9,01369 | 2017-07-21 | 0 | 0 | 0 | 0 | 0 |
| Solanum americanum | GCA_900198685.1_S._americanum_SMRT_RenSeq_HGAP | 8,308 | 2017-07-21 | 0 | 0 | 0 | 0 | 0 |
| Solanum arcanum | GCA_000612985.1_Soarc10 | 665,187 | 2014-03-21 | 20 | 0 | 1 | 0 | 0 |
| Solanum commersonii | GCA_001239805.1_ASM123980v1 | 729,603 | 2015-04-21 | 7 | 0 | 0 | 9 | 0 |
| Solanum habrochaites | GCA_000577655.1_Sohab10 | 724,285 | 2014-02-17 | 18 | 0 | 0 | 0 | 0 |
| Solanum lycopersicum | GCA_000181095.1_ASM18109v1 | 540,589 | 2009-11-06 | 1 | 0 | 3 | 409 | 0 |
| Solanum lycopersicum | GCA_000188115.3_SL3.0 | 828,349 | 2010-12-10 | 0 | 0 | 0 | 4 | 0 |
| Solanum lycopersicum | GCA_000325825.1_S_lycopersicum_mitochondrion_v1.5 | 0,575198 | 2012-02-24 | 0 | 0 | 0 | 0 | 0 |
| Solanum lycopersicum | GCA_900008105.1_V100 | 760,067 | 2014-06-25 | 0 | 0 | 0 | 0 | 0 |
| Solanum lycopersicum | GCA_002954035.1_ASM295403v1 | 824,01 | 2017-11-03 | 0 | 0 | 0 | 0 | 0 |
| Solanum melongena | GCA_000787875.1_SME_r2.5.1 | 833,081 | 2014-09-17 | 0 | 0 | 0 | 0 | 0 |
| Solanum pennellii | GCA_000577875.1_Sopen10 | 720,458 | 2014-02-17 | 16 | 0 | 0 | 0 | 0 |
| Solanum pennellii | GCA_001406875.2_SPENNV200 | 926,426 | 2014-06-25 | 17 | 0 | 0 | 0 | 0 |
| Solanum pennellii | GCA_000820945.1_SPen_1.0 | 720,458 | 2014-09-23 | 16 | 0 | 0 | 0 | 0 |
| Solanum pimpinellifolium | GCA_000230315.1_Sol_pimpi_v1.0 | 688,247 | 2011-10-06 | 71 | 0 | 0 | 0 | 0 |
| Solanum pimpinellifolium | GCA_003660305.1_KAUST_Spi-LA0480_1.0 | 748,694 | 2018-10-15 | 16 | 0 | 0 | 0 | 0 |
| Solanum tuberosum | GCA_000226075.1_SolTub_3.0 | 705,934 | 2011-05-24 | 2 | 0 | 0 | 0 | 0 |
| Solanum tuberosum | GCA_900004685.1_Trinity_Assembly | 90,4582 | 2016-05-15T22:52:00Z | 0 | 0 | 0 | 3 | 0 |
| Solanum verrucosum | GCA_900185145.1_discovar-mp-dt-bn | 730,142 | 2018-05-23 | 424 | 0 | 383 | 3216 | 1 |
| Solanum verrucosum | GCA_900185155.1_10x-bn | 690,417 | 2018-05-23 | 3 | 0 | 0 | 11 | 0 |
| Solanum verrucosum | GCA_900185165.1_falcon-bn | 667,086 | 2018-05-23 | 6 | 0 | 0 | 1 | 0 |
| Solanum verrucosum | GCA_900185175.1_canu-bn | 764,063 | 2018-05-23 | 3 | 0 | 0 | 0 | 0 |
| Solanum verrucosum | GCA_900185185.1_abyss77-mp | 751,585 | 2018-05-23 | 6490 | 0 | 516 | 3846 | 13 |
| Solanum verrucosum | GCA_900185195.1_abyss113-mp | 740,946 | 2018-05-23 | 550 | 0 | 516 | 5024 | 1 |
| Solanum verrucosum | GCA_900185205.1_10x | 688,737 | 2018-05-23 | 4 | 0 | 0 | 10 | 0 |
| Solanum verrucosum | GCA_900185215.1_canu | 722,285 | 2018-05-23 | 1 | 0 | 0 | 1 | 0 |
| Solanum verrucosum | GCA_900185225.1_abyss113 | 730,903 | 2018-05-23 | 435 | 0 | 513 | 5020 | 1 |
| Solanum verrucosum | GCA_900185235.1_abyss77 | 759,168 | 2018-05-23 | 1661 | 0 | 536 | 6127 | 16 |
| Solanum verrucosum | GCA_900185245.1_hgap | 715,934 | 2018-05-23 | 24 | 0 | 0 | 22 | 0 |
| Solanum verrucosum | GCA_900185265.1_discovar-mp-dt | 729,31 | 2018-05-23 | 423 | 0 | 383 | 3216 | 1 |
| Solanum verrucosum | GCA_900185275.1_falcon-dt-bn | 662,264 | 2018-05-23 | 8 | 0 | 0 | 0 | 0 |
| Solanum verrucosum | GCA_900185285.1_falcon | 659,291 | 2018-05-23 | 6 | 0 | 0 | 1 | 0 |
| Solanum verrucosum | GCA_900185295.1_discovar-mp | 728,86 | 2018-05-23 | 423 | 0 | 383 | 3216 | 1 |
| Solanum verrucosum | GCA_900185305.1_hgap-mp | 716,55 | 2018-05-23 | 25 | 0 | 0 | 21 | 0 |
| Solanum verrucosum | GCA_900185315.1_discovar-contig | 710,407 | 2018-05-23 | 427 | 0 | 382 | 3216 | 1 |
| Solanum verrucosum | GCA_900185325.1_falcon-dt | 659,429 | 2018-05-23 | 6 | 0 | 0 | 1 | 0 |
| Solanum verrucosum | GCA_900185335.1_hgap-bn | 749,835 | 2018-05-23 | 25 | 0 | 0 | 21 | 0 |
| Sorghum bicolor | GCA_000003195.3_Sorghum_bicolor_NCBIv3 | 709,345 | 2009-05-22 | 0 | 0 | 0 | 0 | 0 |
| Sorghum bicolor | GCA_000236725.2_ASM23672v2 | 0,018494 | 2011-12-02 | 0 | 0 | 0 | 0 | 0 |
| Sorghum bicolor | GCA_000236745.2_ASM23674v2 | 0,021299 | 2011-12-02 | 0 | 0 | 0 | 0 | 0 |
| Sorghum bicolor | GCA_000236765.2_ASM23676v2 | 0,015475 | 2011-12-02 | 0 | 0 | 0 | 0 | 0 |
| Sorghum bicolor | GCA_003482435.1_Corteva_Sorghum_ONT_TX430_1.0 | 666,155 | 2018-09-06 | 6 | 0 | 0 | 51 | 0 |
| Spinacia oleracea | GCA_000510995.2_Spinach-1.0.3 | 493,772 | 2013-12-16 | 0 | 0 | 0 | 552 | 0 |
| Spinacia oleracea | GCA_002007265.1_ASM200726v1 | 869,946 | 2017-02-27 | 3 | 0 | 1 | 24 | 2 |
| Spirodela polyrhiza | GCA_000504445.1_Spirodela_polyrhiza_v01 | 132,009 | 2013-12-06 | 0 | 0 | 0 | 3 | 0 |
| Spirodela polyrhiza | GCA_001981405.1_ASM198140v1 | 136,67 | 2017-01-17 | 0 | 0 | 0 | 0 | 0 |
| Spirodela polyrhiza | GCA_900536055.1_Sp9509v3 | 142,661 | 2018-08-29 | 0 | 0 | 0 | 99 | 0 |
| Stenocereus thurberi | GCA_002740465.1_Sthu_v1.3 | 853,348 | 2017-10-31 | 0 | 0 | 0 | 0 | 0 |
| Tarenaya hassleriana | GCA_000463585.1_ASM46358v1 | 249,93 | 2013-09-03 | 0 | 0 | 0 | 0 | 0 |
| Tetrabaena socialis | GCA_002891735.1_TetSoc1 | 135,78 | 2018-01-18 | 0 | 0 | 0 | 0 | 0 |
| Tetradesmus obliquus | GCA_900108755.1_sob1 | 107,716 | 2016-11-02 | 0 | 0 | 0 | 0 | 0 |
| Tetradesmus obliquus | GCA_002149895.1_ASM214989v1 | 208,176 | 2017-05-18 | 0 | 0 | 0 | 0 | 0 |
| Theobroma cacao | GCA_000403535.1_Theobroma_cacao_20110822 | 345,994 | 2013-05-29 | 0 | 0 | 0 | 0 | 0 |
| Theobroma cacao | GCA_000208745.2_Criollo_cocoa_genome_V2 | 324,88 | 2016-07-08 | 0 | 0 | 0 | 0 | 0 |
| Thlaspi arvense | GCA_000956625.1_T_arvense_v1 | 343,012 | 2015-03-19 | 0 | 0 | 0 | 103 | 0 |
| Trebouxia gelatinosa | GCA_000818905.1_ASM81890v1 | 61,7262 | 2015-01-15 | 0 | 0 | 0 | 0 | 0 |
| Trebouxia sp. TZW2008 | GCA_002118135.1_TrTZW2008_1.0 | 69,3465 | 2017-03-31T21:29:00Z | 0 | 0 | 0 | 0 | 0 |
| Trebouxiophyceae sp. KSI-1 | GCA_003568905.1_KSI-1_01 | 44,6862 | 2018-08-29 | 55 | 0 | 0 | 7 | 0 |
| Trema orientale | GCA_002914845.1_TorRG33x02_asm01 | 387,958 | 2018-02-02 | 0 | 0 | 0 | 0 | 0 |
| Trifolium medium | GCA_003490085.1_ASM349008v1 | 492,653 | 2018-09-06 | 23 | 0 | 0 | 0 | 0 |
| Trifolium pratense | GCA_000583005.2_Tp1.0 | 304,972 | 2014-02-27 | 2 | 0 | 0 | 0 | 0 |
| Trifolium pratense | GCA_900079335.1_Trpr | 345,991 | 2016-04-12 | 0 | 0 | 0 | 0 | 0 |
| Trifolium subterraneum | GCA_001742945.1_TSUd_r1.1 | 471,834 | 2016-07-16 | 29 | 0 | 1 | 0 | 0 |
| Trifolium subterraneum | GCA_002003065.1_TSUw_r1.0 | 392,71 | 2016-07-26 | 450 | 0 | 3 | 69 | 0 |
| Triticum aestivum | GCA_000210335.1_ASM21033v1 | 1,26608 | 2010-07-15 | 0 | 0 | 0 | 0 | 0 |
| Triticum aestivum | GCA_000188135.1_Te5A_1.0 | 159,087 | 2011-02-04 | 1 | 0 | 0 | 2 | 0 |
| Triticum aestivum | GCA_000334095.1_LCG | 3800,33 | 2012-12-23 | 1418 | 0 | 2 | 20 | 1 |
| Triticum aestivum | GCA_000334135.1_OA | 437,106 | 2013-01-09 | 0 | 0 | 0 | 78 | 0 |
| Triticum aestivum | GCA_001077335.1_ASM107733v1 | 58,5022 | 2014-07-28 | 0 | 0 | 0 | 0 | 0 |
| Triticum aestivum | GCA_900000045.1_Synthetic_W7984_assembly | 9134,02 | 2014-11-01 | 1638 | 0 | 3 | 17021 | 23 |
| Triticum aestivum | GCA_000818885.1_ASM81888v1 | 65,1111 | 2015-01-02 | 0 | 0 | 0 | 2 | 0 |
| Triticum aestivum | GCA_001485685.1_Hall1 | 44,4015 | 2015-11-19 | 5 | 0 | 0 | 0 | 0 |
| Triticum aestivum | GCA_900067645.1_Triticum_aestivum_CS42_TGAC_v1 | 13427,4 | 2016-01-14 | 330 | 0 | 0 | 4 | 0 |
| Triticum aestivum | GCA_001889205.1_ASM188920v1 | 0,942499 | 2016-11-03 | 5 | 0 | 29 | 198 | 0 |
| Triticum aestivum | GCA_001889245.1_ASM188924v1 | 2,42226 | 2016-11-03 | 8 | 0 | 34 | 498 | 0 |
| Triticum aestivum | GCA_002158495.1_ASM215849v1 | 567,21 | 2017-05-08 | 0 | 0 | 0 | 0 | 0 |
| Triticum aestivum | GCA_002220415.2_Triticum_3.1 | 15344,7 | 2017-07-13 | 180 | 0 | 0 | 7 | 0 |
| Triticum aestivum | GCA_002780475.1_Can7DL_1.0 | 240,199 | 2017-11-16 | 0 | 0 | 0 | 8 | 0 |
| Triticum aestivum | GCA_002780545.1_NS1v1 | 609,488 | 2017-11-16 | 4 | 0 | 0 | 0 | 0 |
| Triticum aestivum | GCA_002780565.1_NS2v1 | 564,3 | 2017-11-16 | 2 | 0 | 0 | 11 | 0 |
| Triticum aestivum | GCA_900236235.1_cs42_chr3DL | 452,948 | 2017-12-01 | 3 | 0 | 0 | 45 | 0 |
| Triticum aestivum | GCA_900241085.1_wheat_TGACv2 | 13916,9 | 2018-02-14 | 184 | 0 | 0 | 0 | 0 |
| Triticum aestivum | GCA_002999095.1_7DS_MTP_mate-pair_complete | 574,295 | 2018-03-14 | 0 | 0 | 0 | 0 | 0 |
| Triticum aestivum | GCA_900519105.1_iwgsc_refseqv1.0 | 14547,3 | 2018-08-19 | 0 | 0 | 0 | 0 | 0 |
| Triticum dicoccoides | GCA_002162155.1_WEWSeq_v.1.0 | 10494,9 | 2017-05-18 | 46 | 0 | 0 | 11 | 0 |
| Triticum dicoccoides | GCA_900184675.1_WEW_v1 | 10495 | 2017-06-09 | 46 | 0 | 3 | 21 | 0 |
| Triticum dicoccoides | GCA_900323565.1_Tdic5A_chr | 353,054 | 2018-05-26 | 5 | 0 | 0 | 0 | 0 |
| Triticum dicoccoides | GCA_900323575.1_Tdic7B_chr | 585,067 | 2018-05-26 | 18 | 0 | 0 | 4 | 0 |
| Triticum dicoccoides | GCA_900323585.1_Tdic2A_chr | 288,316 | 2018-05-26 | 1 | 0 | 0 | 6 | 0 |
| Triticum dicoccoides | GCA_900323595.1_Tdic3A_chr | 431,061 | 2018-05-26 | 1 | 0 | 0 | 0 | 0 |
| Triticum dicoccoides | GCA_900323605.1_Tdic7A_chr | 497,42 | 2018-05-26 | 4 | 0 | 0 | 0 | 0 |
| Triticum dicoccoides | GCA_900323615.1_Tdic2B_chr | 477,083 | 2018-05-26 | 6 | 0 | 0 | 5 | 0 |
| Triticum dicoccoides | GCA_900323625.1_Tdic4B_chr | 518,951 | 2018-05-26 | 19 | 0 | 0 | 3 | 0 |
| Triticum dicoccoides | GCA_900323635.1_Tdic1A_chr | 434,779 | 2018-05-26 | 4 | 0 | 0 | 3 | 0 |
| Triticum dicoccoides | GCA_900323645.1_Tdic6A_chr | 392,684 | 2018-05-26 | 0 | 0 | 0 | 5 | 0 |
| Triticum dicoccoides | GCA_900323655.1_Tdic6B_chr | 624,303 | 2018-05-26 | 7 | 0 | 0 | 1 | 0 |
| Triticum dicoccoides | GCA_900323665.1_Tdic5B_chr | 441,177 | 2018-05-26 | 10 | 0 | 0 | 1 | 0 |
| Triticum dicoccoides | GCA_900323675.1_Tdic4A_chr | 388,595 | 2018-05-26 | 4 | 0 | 0 | 8 | 0 |
| Triticum dicoccoides | GCA_900323685.1_Tdic1B_chr | 700,417 | 2018-05-26 | 11 | 0 | 0 | 0 | 0 |
| Triticum dicoccoides | GCA_900323695.1_Tdic3B_chr | 552,916 | 2018-05-26 | 5 | 0 | 0 | 1 | 0 |
| Triticum urartu | GCA_000347455.1_ASM34745v1 | 3747,05 | 2013-03-01 | 600 | 0 | 0 | 3563 | 0 |
| Triticum urartu | GCA_003073215.1_Tu2.0 | 4851,9 | 2018-04-26 | 3 | 0 | 0 | 4 | 0 |
| Ulva mutabilis | GCA_900538255.1_Ulvmu_WT_fa | 98,4847 | 2018-09-12 | 2 | 0 | 0 | 0 | 0 |
| uncultured Bathycoccus | GCA_000259855.1_ASM25985v1 | 5,18308 | 2011-10-31 | 0 | 0 | 0 | 0 | 0 |
| Urochloa ruziziensis | GCA_003016355.1_Bruz | 732,531 | 2018-03-21 | 0 | 0 | 0 | 0 | 0 |
| Utricularia gibba | GCA_002189035.1_U_gibba_v2 | 100,689 | 2017-05-31 | 0 | 0 | 0 | 0 | 0 |
| Vaccinium macrocarpon | GCA_000775335.2_ASM77533v2 | 414,622 | 2014-09-04 | 1 | 0 | 0 | 4 | 0 |
| Vicia faba | GCA_001375635.1_VfEP_Reference-Unigene | 80,3627 | 2015-03-24 | 0 | 0 | 1 | 15 | 0 |
| Vigna angularis | GCA_001190045.1_Vigan1.1 | 467,301 | 2015-07-30 | 3 | 0 | 0 | 219 | 0 |
| Vigna angularis var. angularis | GCA_000465365.1_Vigna_angularis | 291,824 | 2013-09-06 | 0 | 0 | 0 | 0 | 0 |
| Vigna angularis var. angularis | GCA_001723775.1_ASM172377v1 | 444,439 | 2016-09-13 | 1 | 0 | 1 | 1 | 0 |
| Vigna radiata | GCA_000180895.1_ASM18089v1 | 10,1012 | 2009-11-30 | 2 | 0 | 0 | 7 | 0 |
| Vigna radiata var. radiata | GCA_000741045.2_Vradiata_ver6 | 463,638 | 2014-08-18 | 0 | 0 | 0 | 4 | 0 |
| Vigna radiata var. radiata | GCA_001584445.1_ASM158444v1 | 454,907 | 2016-03-09 | 0 | 0 | 0 | 0 | 0 |
| Vigna unguiculata subsp. unguiculata | GCA_001687525.1_Cowpea_0.03 | 695,046 | 2016-07-20 | 14 | 0 | 0 | 1114 | 1 |
| Viola pubescens var. scabriuscula | GCA_002752925.1_violet_k79_twolib_greater_than_500bp | 318,366 | 2017-11-06 | 0 | 0 | 0 | 2 | 0 |
| Vitis aestivalis | GCA_001562795.1_VitisNorton_MSU1.0 | 432,755 | 2016-02-17 | 2 | 0 | 0 | 1 | 0 |
| Vitis cinerea x Vitis riparia | GCA_001282645.1_BoeWGS1.0 | 539,624 | 2015-06-30 | 0 | 0 | 0 | 0 | 0 |
| Vitis vinifera | GCA_000003745.2_12X | 486,197 | 2007-02-13 | 0 | 0 | 0 | 1 | 0 |
| Vitis vinifera | GCA_002922885.1_Chkhaveri_assembly01 | 427,171 | 2017-06-14 | 0 | 0 | 0 | 0 | 0 |
| Vitis vinifera | GCA_002923015.1_Saperavi_assembly01 | 427,04 | 2017-06-22 | 0 | 0 | 0 | 0 | 0 |
| Vitis vinifera | GCA_002923105.1_Meskhetian_assembly01 | 427,211 | 2017-06-22 | 0 | 0 | 0 | 0 | 0 |
| Vitis vinifera | GCA_002923165.1_Rkatsiteli_assembly01 | 426,616 | 2017-06-22 | 0 | 0 | 0 | 0 | 0 |
| Volvox carteri f. nagariensis | GCA_000143455.1_v1.0 | 137,684 | 2010-07-06 | 0 | 0 | 0 | 4 | 0 |
| Xanthoceras sorbifolium | GCA_003430845.1_ASM343084v1 | 504,383 | 2018-08-28 | 1 | 0 | 0 | 1 | 0 |
| Xerophyta viscosa | GCA_002076135.1_ASM207613v1 | 295,462 | 2017-03-31 | 0 | 0 | 0 | 0 | 0 |
| Yamagishiella unicocca | GCA_003116995.1_YamagishiellaPlus_1.0 | 134,235 | 2018-04-06T20:58:00Z | 0 | 0 | 0 | 0 | 0 |
| Yamagishiella unicocca | GCA_003117035.1_YamagishiellaMinus_1.0 | 140,837 | 2018-04-06T21:09:00Z | 0 | 0 | 0 | 0 | 0 |
| Zea mays | GCA_000005005.6_B73_RefGen_v4 | 2135,08 | 2010-01-29 | 0 | 0 | 0 | 0 | 0 |
| Zea mays | GCA_000223545.1_ZeaMays_PT_EDMX2233_1.0 | 177,051 | 2011-08-16 | 14 | 0 | 0 | 23 | 0 |
| Zea mays | GCA_000275765.1_ZmaysB73_wgs_1.0 | 1,33507 | 2012-07-03 | 0 | 0 | 0 | 0 | 0 |
| Zea mays | GCA_003185045.1_Zm-Mo17-REFERENCE-CAU-1.0 | 2182,61 | 2018-06-04 | 0 | 0 | 0 | 0 | 0 |
| Zea mays | GCA_003704525.1_Zm-PE0075-REFERENCE-TUM-1.0 | 2198,5 | 2018-10-31 | 0 | 0 | 0 | 0 | 0 |
| Zea mays | GCA_003709335.1_Zm-DK105-REFERENCE-TUM-1.0 | 2288,19 | 2018-10-31 | 0 | 0 | 0 | 0 | 0 |
| Zea mays subsp. mays | GCA_001644905.2_Zm-W22-REFERENCE-NRGENE-2.0 | 2133,88 | 2016-05-11 | 0 | 0 | 0 | 0 | 0 |
| Zea mays subsp. mays | GCA_001984235.2_Zm-EP1-REFERENCE-TUM-1.0 | 2455,26 | 2017-02-03 | 53 | 0 | 0 | 1085 | 0 |
| Zea mays subsp. mays | GCA_001990705.1_Zm-F7-REFERENCE-TUM-1.0 | 2392,8 | 2017-02-03 | 24 | 0 | 0 | 1300 | 0 |
| Zea mays subsp. mays | GCA_002237485.1_Zm-PH207-REFERENCE_NS-UIUC_UMN-1.1 | 2155,82 | 2017-07-28 | 7 | 0 | 0 | 1 | 0 |
| Zea mays subsp. mays | GCA_002682915.2_Zm_CML247_REFERENCE_PANZEA.1.2 | 2197,97 | 2017-10-23 | 0 | 0 | 0 | 0 | 0 |
| Zea mays subsp. mays | GCA_002813505.1_ASM281350v1 | 2041,55 | 2017-12-06 | 14 | 0 | 0 | 4 | 0 |
| Zea mays subsp. mexicana | GCA_002813485.1_ASM281348v1 | 1204,28 | 2017-12-06 | 4 | 0 | 0 | 2383 | 0 |
| Zizania latifolia | GCA_000418225.1_Zizania_latifolia_v01 | 603,989 | 2013-07-03 | 0 | 0 | 0 | 1 | 0 |
| Ziziphus jujuba | GCA_000826755.1_ZizJuj_1.1 | 437,754 | 2014-10-24 | 1 | 0 | 0 | 27 | 0 |
| Ziziphus jujuba | GCA_001835785.1_ASM183578v1 | 351,097 | 2016-10-24 | 0 | 0 | 0 | 0 | 0 |
| Zostera marina | GCA_001185155.1_Zosma_marina.v.2.1 | 203,914 | 2015-07-22 | 0 | 0 | 0 | 0 | 0 |
| Zoysia japonica | GCA_001602275.1_ASM160227v1 | 334,384 | 2016-03-15 | 0 | 0 | 0 | 7 | 0 |
| Zoysia matrella | GCA_001602295.1_ASM160229v1 | 563,439 | 2016-03-15 | 0 | 0 | 0 | 0 | 0 |
| Zoysia pacifica | GCA_001602315.1_ASM160231v1 | 397,01 | 2016-03-15 | 0 | 0 | 0 | 0 | 0 |

Table S2: A selection of taxonomic profilers and binners available for analysis of metagenomic datasets.

|  | Model-based | K-mer based | Mapping based |
| --- | --- | --- | --- |
| Taxonomic Profilers | Taxy-pro (10.1093/bioinformatics/btt077) | CLARK (10.1186/s12864-015-1419-2)  Metapalette (10.1128/mSystems.00020-16)  FOCUS (10.7717/peerj.425) | TIPP (Cami) (43)  MetaPhlAn2 ([10.1038/nmeth.3589](http://dx.doi.org/10.1038/nmeth.3589))  MetaPhyler (10.1186/1471-2164-12-S2-S4)  DUDes (10.1093/bioinformatics/btw150)  Constrains (10.1038/nbt.3319) |
| Taxonomic Binners |  | Phylopythia S+ (10.7717/peerj.1603)  Kraken ([dx.doi.org/10.1186/gb-2014-15-3-r46](http://dx.doi.org/10.1186/gb-2014-15-3-r46))  Latent Strain Analysis (10.1038/nbt.3319)  Genomic Origins Through Taxonomic CHAllenge (GOTTCHA) (10.1093/nar/gkv180) | MIDAS (10.1101/gr.201863.115)  PanPhlAn (10.1038/nmeth.3802)  Taxator-tk (10.1093/bioinformatics/btu745)  Genometa (10.1371/journal.pone.0041224)  MEGAN CE (10.1371/journal.pcbi.1004957) |

Table S3: Recommended tools for genome binning workflow. Tools for assembly and binning are recommended based on performance in the Cami challenge (Sczyrba et al., 2017).

| Workflow Step | Tool |  |
| --- | --- | --- |
| Quality evaluation | FastQC (http://www.bioinformatics.babraham.ac.uk/projects/fastqc/) |  |
| Read trimming | Trimmomatic (10.1093/bioinformatics/btu170) |  |
| Assembly | Megahit (doi: 10.1093/bioinformatics/btv033, 10.1016/j.ymeth.2016.02.020)  Minia (https://github.com/GATB/minia)  Meraculous (10.1371/journal.pone.0023501) |  |
| Assembly evaluation | MetaQUAST (10.1093/bioinformatics/btv697) |  |
| Read mapping | BWA (doi: 10.1093/bioinformatics/btp324)  bbmap (http://sourceforge. net/projects/bbmap)  Bowtie2 (doi: 10.1038/nmeth.1923) |  |
| Binning | MaxBin2.0 (10.1093/bioinformatics/btv638)  Metabat (10.7717/peerj.1165)  MetaWatt (10.3389/fmicb.2012.00410) |  |
| Bin evaluation and classification | CheckM (10.1101/gr.186072.114)  Phyla-AMPHORA (doi: 10.1093/molbev/mst059)  GTDB-Tk (https://github.com/Ecogenomics/GtdbTk)  dRep (doi: 10.1038/ismej.2017.126)  Anvi’o (<http://merenlab.org/software/anvio/>; doi: 10.7717/peerj.1319) |  |
| Composite Workflow | Atlas 2 (https://metagenome-atlas.readthedocs.io/en/latest/) | |
| Online annotation of bins | RAST (http://rast.nmpdr.org/)  IMG4 (https://img.jgi.doe.gov/)  MaGE (https://www.genoscope.cns.fr/agc/microscope/) |  |
